# Meningeal lymphatic vessels regulate brain tumor drainage and immunity

**DOI:** 10.1038/s41422-020-0287-8

**Published:** 2020-02-24

**Authors:** Xueting Hu, Qiuping Deng, Lu Ma, Qingqing Li, Yidong Chen, Yuhan Liao, Fan Zhou, Chen Zhang, Linlin Shao, Jun Feng, Tubao He, Weihai Ning, Yan Kong, Yingqing Huo, Aibin He, Bing Liu, Jingjing Zhang, Ralf Adams, Yulong He, Fuchou Tang, Xiuwu Bian, Jincai Luo

**Affiliations:** 1 Institute of Molecular Medicine, Beijing Key Laboratory of Cardiometabolic Molecular Medicine, Peking University, 100871 Beijing, China; 2 Biodynamic Optical Imaging Center, College of Life Sciences, Peking University, 100871 Beijing, China; 3 State Key Laboratory of Proteomics, Translational Medicine Center of Stem Cells, 307-Ivy Translational Medicine Center, Laboratory of Oncology, Affiliated Hospital, Academy of Military Medical Sciences, 100071 Beijing, China; 4 Department of Neurobiology, School of Basic Medical Sciences, Capital Medical University, 100069 Beijing, China; 5 Eye Hospital of China Academy of Chinese Medical Sciences, 100040 Beijing, China; 6 Sanbo Brain Hospital, Capital Medical University, 100093 Beijing, China; 7 Key Laboratory of Carcinogenesis and Translational Research (Ministry of Education), Department of Cell Biology, Peking University Cancer Hospital and Institute, 100142 Beijing, China; 8 Peking-Tsinghua Center for Life Sciences, 100871 Beijing, China; 9 Affiliated Hospital of Guangdong Medical University, Zhanjiang, 524001 Guangdong China; 10 Max-Planck-Institute for Molecular Biomedicine, Department of Tissue Morphogenesis, and University of Münster, Faculty of Medicine, Münster, D-48149 Germany; 11 Cyrus Tang Hematology Center, Collaborative Innovation Center of Hematology, Soochow University, Suzhou, 215123 Jiangsu China; 12 Institute of Pathology and Southwest Cancer Center, Key Laboratory of the Ministry of Education, Southwest Hospital, Army Medical University (Third Military Medical University), 400038 Chongqing, China

**Keywords:** Cancer, Immunology, Cell biology

## Abstract

Recent studies have shown that meningeal lymphatic vessels (MLVs), which are located both dorsally and basally beneath the skull, provide a route for draining macromolecules and trafficking immune cells from the central nervous system (CNS) into cervical lymph nodes (CLNs), and thus represent a potential therapeutic target for treating neurodegenerative and neuroinflammatory diseases. However, the roles of MLVs in brain tumor drainage and immunity remain unexplored. Here we show that dorsal MLVs undergo extensive remodeling in mice with intracranial gliomas or metastatic melanomas. RNA-seq analysis of MLV endothelial cells revealed changes in the gene sets involved in lymphatic remodeling, fluid drainage, as well as inflammatory and immunological responses. Disruption of dorsal MLVs alone impaired intratumor fluid drainage and the dissemination of brain tumor cells to deep CLNs (dCLNs). Notably, the dendritic cell (DC) trafficking from intracranial tumor tissues to dCLNs decreased in mice with defective dorsal MLVs, and increased in mice with enhanced dorsal meningeal lymphangiogenesis. Strikingly, disruption of dorsal MLVs alone, without affecting basal MLVs or nasal LVs, significantly reduced the efficacy of combined anti-PD-1/CTLA-4 checkpoint therapy in striatal tumor models. Furthermore, mice bearing tumors overexpressing VEGF-C displayed a better response to anti-PD-1/CTLA-4 combination therapy, and this was abolished by CCL21/CCR7 blockade, suggesting that VEGF-C potentiates checkpoint therapy via the CCL21/CCR7 pathway. Together, the results of our study not only demonstrate the functional aspects of MLVs as classic lymphatic vasculature, but also highlight that they are essential in generating an efficient immune response against brain tumors.

## Introduction

The concept of immune privilege in the central nervous system (CNS) has been substantially reconsidered in the past decade.^1^ This concept was proposed more than half a century ago based on the experimental findings that foreign tissue grafts including tumors in the brain parenchyma were not rejected by the immune system of the host.^2,3^ Indeed, the CNS has unique anatomical features such as the blood–brain barrier and a lack of classic lymphatic vessels (LVs) in the parenchyma. However, evidence has emerged that CNS-derived soluble molecules and lymphocytes circulate through the healthy brain, and extensive cross-talk between the brain and the peripheral immune system exists under pathological conditions such as autoimmunity and infection.^4^ To visualize this process, fluorescent tracer dye was injected into the gray matter from where it migrates out to the cervical lymph nodes (CLNs).^5^ Subsequently, using a polarized macroscopic system, convective fluid fluxes with rapid interchange between cerebrospinal fluid (CSF) and interstitial fluid in the parenchyma were reported and named the “glymphatic” system based on its functional similarity to the lymphatic system in peripheral tissue, and on the important role of glial aquaporin-4 channels in the convective fluid transport.^6^ In searching for the pathways of immune cell movement and CSF drainage throughout the meninges, a lymphatic network in the dura mater situated bilaterally along the superior sagittal sinus (SSS) and transverse sinus (TS) has been recently rediscovered in rodents^7,8^ and humans,^9^ and has been shown to possess some of the classic lymphatic functions. These studies indicate that the immune system functions in some sub-regions of the CNS similarly to in the periphery.

The features of meningeal LVs (MLVs) have been characterized and their functions in brain physiology have been gradually demonstrated. Like conventional lymphatic endothelial cells (LECs), meningeal LECs (MLECs) express the markers of CD31, VEGFR3, Prox1, PDPN, LYVE-1, and CCL21; and they efficiently drain both soluble molecules and immune cells from the subarachnoid space into CLNs.^7,8^ By injection of macromolecules, T cells, and dendritic cells (DCs) into the CSF, MLVs were shown to be capable of carrying CSF components and immune cells from the CNS to the CLNs in a physiological manner.^10–12^ Pathologically, while ablation of MLVs reduced the inflammatory responses in an animal model of multiple sclerosis,^10^ dysfunction of MLVs was shown to be an aggravating factor in the pathology of Alzheimer’s disease and age-associated cognitive decline.^11,13^ Together, MLVs play an important role in connecting the CNS to peripheral immunity and thus represent a promising therapeutic target for neurodegenerative and neuroinflammatory diseases. In contrast to the above findings that were obtained with the MLVs located at the dorsal part of the skull,^7,8^ several recent studies support the role of the basal MLVs in drainage of fluid from the CNS.^14–16^ Consistently, a very recent study showed that the MLVs located at the basal part of the skull appeared to be morphologically distinct and have stronger draining functions than dorsal MLVs.^11^ Therefore, the structural and functional relationships and inter-dependence of these MLVs need to be further investigated.

Brain tumors, including primary and metastatic tumors, are among the most feared of all forms of cancer with very limited treatment options and a poor prognosis.^17–19^ Malignant cerebral gliomas, the most common primary tumor in the CNS, comprising ~80% of all malignant primary brain tumors,^20^ have a strong tendency to infiltrate.^21^ Nevertheless, metastatic brain tumors are the most frequently occurring intracranial tumors.^22^ It is well known that, among common cancers, metastatic melanoma has the highest risk of spreading to the CNS; it has a marked propensity to metastasize to the brain where it is characterized by meningeal and ventricular metastases.^18^ For decades it has been known that brain tumors can elicit potent antitumor immune responses. Specifically, using animal models, it has been demonstrated that soluble tumor antigens within the CNS can reach the CLNs via CSF drainage to stimulate specific T cells.^23^ Function-blocking antibodies against PD-1 and CTLA-4 have been shown to enhance antitumor T cell responses and resulted in durable antitumor activity in several mouse intracranial tumor models and clinical trials in patients.^24–27^ However, the response of brain tumors to immunotherapy is relatively weak and survival is still poor, suggesting that more attention should be paid to understand the immunity in brain tumors. Tumor-associated lymphatics are critical for the drainage of intratumor fluid, macromolecules (antigens), metastasis, and immunity.^28–31^ The role of MLVs linking the CNS to peripheral immunity prompted us to study their involvement in the fluid drainage of brain tumors and immunity. One intriguing question is whether MLVs act as a route out of the tumor that can be used for fluid drainage and tumor dissemination. In addition, it is important to determine whether and how MLVs participate in tumor immune responses, although MLVs within the dura mater are not physically close to tumors in the parenchyma.

To investigate the roles of MLVs in the fluid drainage of brain tumors and immunity, we developed two mouse models with GL261 or B16 cells injected beneath the dura or in the striatum. We found that intracranial tumors induced extensive remodeling of dorsal MLVs, while basal MLVs underwent only mild remodeling at the late stage of tumor progression. Consistently, we found that specific pharmacochemical ablation of dorsal MLVs significantly blocked DC trafficking from brain tumors to dCLNs, and reduced the efficacy of anti-PD-1/CTLA-4 combination therapy. Mechanistically, we demonstrated that VEGF-C mediated the potentiation of immunotherapy and this was dependent on CCL21/CCR7 signaling. Our findings demonstrate that the dorsal MLVs play an essential role in brain tumor immunity and might be targeted in brain tumor immunotherapy.

## Results

### Intracranial tumors induce remodeling of dorsal MLVs

**Fig. 1:**
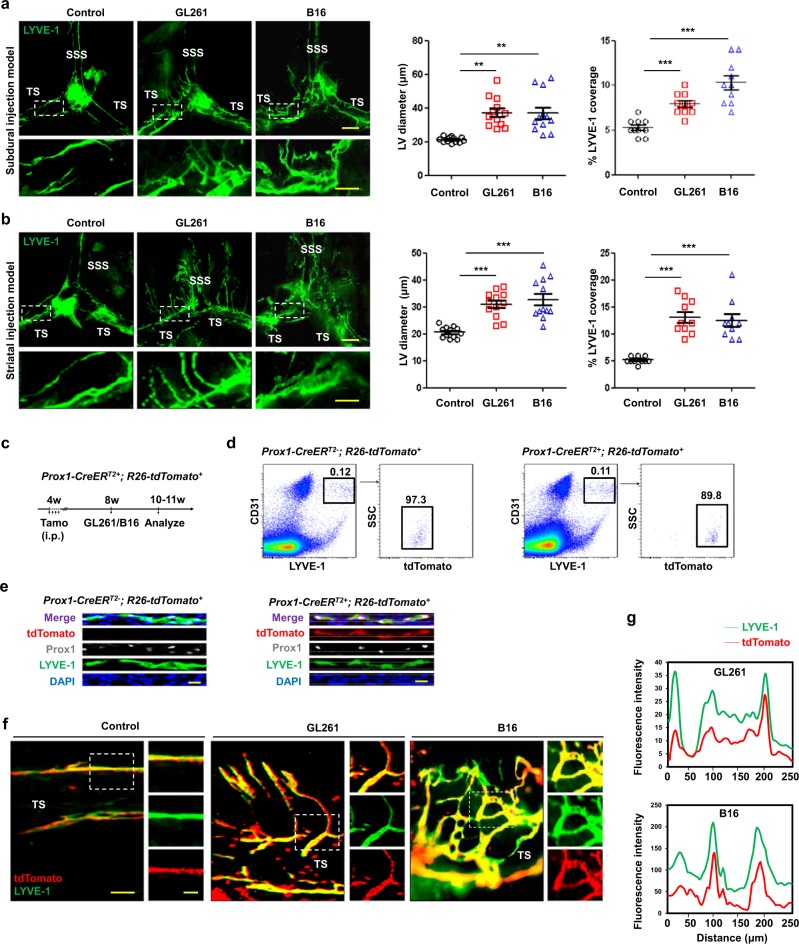
Brain tumors induce dorsal meningeal lymphangiogenesis. **a, b** Left, Representative meningeal LYVE-1 staining 1 week after subdural injection (**a**) and 2 weeks after striatal injection (**b**) of GL261 or B16 cells into WT mice (SSS superior sagittal sinus, TS transverse sinus). Right, Quantification of the diameter (*n* = 12) and percentage area (*n* = 10) of LYVE-1^+^ MLVs around the TS. Scale bars, 500 µm in wide-fields; 100 µm in insets. **c** Schematic diagram of tamoxifen administration and tissue analysis schedule in *Prox1-Cre*^*ERT2+*^*; R26-tdTomato*^*+*^ mice. **d** Representative FACS plots and gating scheme of CD31 + LYVE-1^+^tdTomato^+^ MLECs isolated from normal *Prox1-Cre*^*ERT2*−^; *R26-tdTomato*^*+*^ and *Prox1-Cre*^*ERT2+*^; *R26-tdTomato*^*+*^ mice 3 weeks after tamoxifen induction. **e** Images of Prox1, LYVE-1 staining and tdTomato signals in the TS of meninges from *Prox1-Cre*^*ERT2*−^*; R26-tdTomato*^*+*^ and *Prox1-Cre*^*ERT2+*^*; R26-tdTomato*^*+*^ mice 3 weeks after tamoxifen induction. Scale bars, 20 µm. **f** LYVE-1 staining of MLVs around the TS in *Prox1-Cre*^*ERT2+*^; *R26-tdTomato*^*+*^ mice 2 weeks after subdural injection of GL261 or B16 cells. Scale bars, 100 µm in wide-fields; 50 µm in insets. **g** Co-localization analysis of tdTomato and LYVE-1 in the insets shown in **f**. Data are presented as means ± SEM; each symbol represents an individual mouse. **P < 0.01, ***P < 0.001; two-way ANOVA (**a**, **b**). Data are from at least three independent experiments (**a**–**g**).

Local lymphatic remodeling plays complex and important roles in tumor progression.^32^ To determine whether intracranial tumors are capable of inducing meningeal lymphangiogenesis, we first subdurally injected GL261 glioma cells or B16 melanoma cells, which are both syngeneic to C57BL/6 mice, into C57BL/6 mice (Supplementary information, Fig. S1a). We then investigated the changes of MLVs in mice bearing GL261 or B16 tumors using LYVE-1 immunostaining and found significant lymphatic remodeling, especially in the dorsal meninges 1 week after tumor cell injection (Fig. 1a). We observed clearly dilated vessels positive for LYVE-1 as well as a greater LYVE-1^+^ vessel area along the sinuses in the meninges, especially around the TS (Fig. 1a). Quantification of LYVE-1^+^ vessel diameter and area confirmed lymphangiogenesis in the meninges of tumor-bearing mice (Fig. 1a). Next, we injected GL261 or B16 tumor cells into the striatum of mice (Supplementary information, Fig. S1b). Similarly, dorsal MLV remodeling (widening and sprouting) occurred 2 weeks after tumor injection, and thereafter the MLV network expanded along the TS and SSS in these mice but not in control mice (Fig. 1b).

The tumors associated with lymphangiogenesis have various LEC origins, such as pre-existing LECs or bone marrow-derived lymphatic progenitor cells.^33^ Since MLECs display a relatively unique signature,^10^ we wondered whether meningeal lymphangiogenesis is derived from different origins. To test this, we used inducible Prox1^+^ LEC-specific lineage-tracing *Prox1-CreER*^*T2+*^*; Rosa26-tdTomato*^*+*^ mice (Fig. 1c). Three weeks after tamoxifen administration, ≥ 89% of the LYVE-1^+^ MLECs expressed tdTomato, indicating efficient targeting by the *Prox1-Cre* transgene (Fig. 1d). In addition, immunostaining for Prox1 and LYVE-1 showed that tdTomato was faithfully expressed in MLECs (Fig. 1e). Whole-mount staining of the MLVs around the TS showed that the expression of LYVE-1 in sprouting MLVs was mostly co-localized with tdTomato (Fig. 1f, g), suggesting that meningeal lymphangiogenesis is at least partially attributable to the sprouting of pre-existing MLECs.

Given the very recent study of basal MLVs,^11^ we wondered whether they also undergo remodeling in response to intracranial tumors. Interestingly, lymphangiogenesis was not evident in basal MLVs even 3 weeks after tumor cell inoculation into the striatum. Quantitation of LYVE-1^+^ vessels revealed a slight increase in their area in 4 weeks (Supplementary information, Fig. S2a). Besides MLV systems, previous reports have suggested that the nasal LVs also contribute to CSF drainage and undergo remodeling in the experimental autoimmune encephalomyelitis-induced neuroinflammation model.^10,12^ However, no changes in the nasal LVs were found in 4 weeks in mice bearing striatal tumors (Supplementary information, Fig. S2b). Notably, our results showed that dorsal MLVs underwent extensive remodeling 2 weeks after tumor inoculation into the striatum (Fig. 1b). These results suggest that dorsal MLVs undergo extensive remodeling in response to brain tumors, whereas basal MLVs and nasal LVs are relatively less sensitive.

### Dorsal MLVs mediate intratumor fluid drainage and the dissemination of intracranial tumor cells to CLNs

**Fig. 2:**
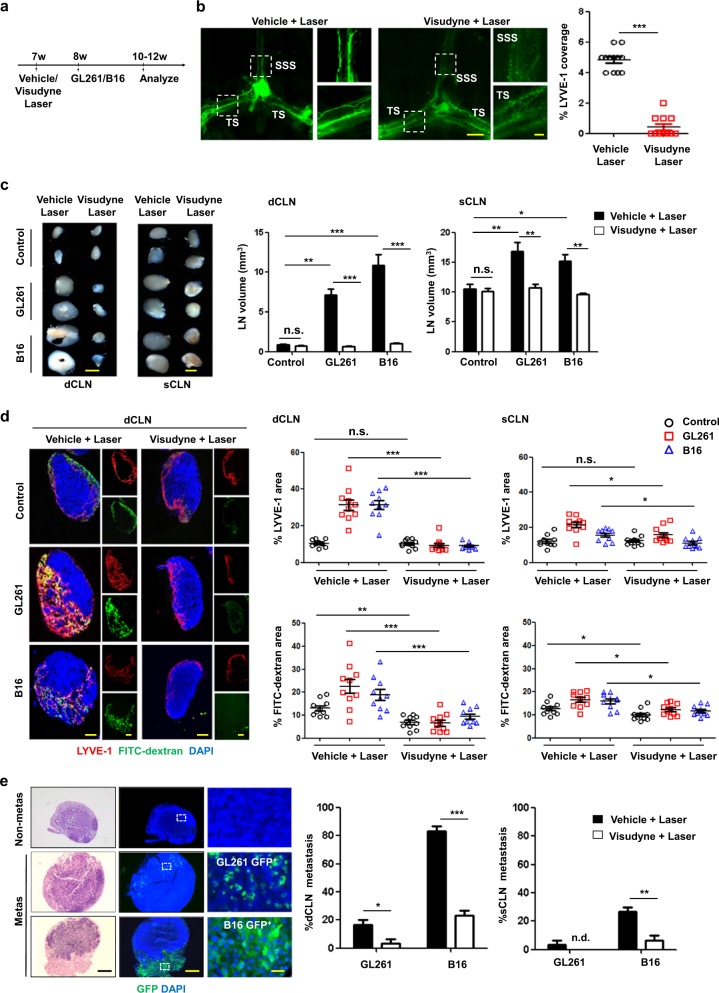
Dorsal meningeal lymphatic vasculature mediates the spread of brain tumor cells to CLNs. **a** Protocol: 7-week-old WT mice were treated with Vehicle + Laser or Visudyne + Laser (to ablate dorsal MLVs), and 1 week later GL261 or B16 cells were injected into the striatum. **b** Left panels, representative meningeal LYVE-1 staining 1 week after mice were treated with Vehicle + Laser or Visudyne + Laser (scale bars, 500 µm in wide-fields; 100 µm in insets). Right panel, quantification of the percentage area of LYVE-1 (*n* = 12). **c** Left panels, representative images of dCLNs and sCLNs 2 weeks after striatal injection of GL261 or B16 cells into mice treated with Vehicle + Laser or Visudyne + Laser (scale bars, 1 mm). Right panels, quantification of the volumes of dCLNs and sCLNs (*n* = 12). **d** Left panels, representative sections of dCLNs showing DAPI, LYVE-1, and FITC-dextran staining 30 min after injection of FITC-dextran into tumors of mice (same site in control mice) treated with Vehicle + Laser or Visudyne + Laser (scale bars, 300 µm). Right panels, quantification of the fraction of LYVE-1 and FITC-dextran area in dCLNs and sCLNs (*n* = 10). **e** Left panels, histology of representative dCLNs containing tumor cell metastases in mice after i.c.m. injection of GL261-GFP^+^ or B16-GFP^+^ cells. Fluorescence micrographs of parallel sections reveal strong GFP expression by metastatic tumor cells. Images on the right correspond to the insets (Non-metas, non metastatic; metas, metastatic; scale bars, 500 µm in wide-fields, 50 µm in insets). Right panels, percentages of GFP^+^ dCLNs and sCLNs (*n* = 20; n.d. not detected). Data are presented as means ± SEM. *P < 0.05, **P < 0.01, ***P < 0.001, n.s. not significant; two-tailed unpaired Student’s *t* test (**b**), or two-way ANOVA (**c–e**). Data are from at least three independent experiments (**a**–**e**).

To assess the role of the dorsal meningeal lymphatic vasculature in brain tumor progression, we used a pharmacochemical approach to directly ablate the dorsal MLVs. By injecting visudyne, which has been shown to efficiently ablate MLVs with a nonthermal 689-nm laser,^10^ into the cisterna magna of wild-type (WT) mice, MLV-defective mice (Visudyne + Laser) were generated. Mice injected with the vehicle followed by laser treatment served as MLV-intact controls (Vehicle + Laser). This approach resulted in effective ablation of MLVs around the SSS and TS (Fig. 2a, b), and no differences were detected around the basal MLVs or nasal LVs (Supplementary information, Fig. S3a, b) between the MLV-intact and MLV-defective mice, showing that this method selectively ablated the dorsal MLVs. In addition, we found that the ablation of dorsal MLVs did not affect the meningeal blood vessels (Supplementary information, Fig. S4a), consistent with the previous report.^10^ Then we injected GL261 cells or B16 cells into the striatum of MLV-intact and MLV-defective mice, using PBS injection as control. We found that ablation of the dorsal MLVs affected neither tumor angiogenesis nor tumor growth (Supplementary information, Fig. S4b, c). Interestingly, MRI imaging showed that MLV-defective mice displayed aggravated cerebral edema in the parenchyma as indicated by a strong MRI signal change compared with MLV-intact mice (Supplementary information, Fig. S4d), suggesting the involvement of dorsal MLVs in regulation of intratumor drainage. We further examined the relationship between the integrity of MLVs and the size of CLNs in mice with intracranial tumors. Basically, MLV-defective mice without tumors did not display changes in CLN volume (Fig. 2c). We found that the sizes of dCLNs were markedly greater (GL261, ~7.91-fold; B16, ~12.04-fold), and the sizes of superficial CLNs (sCLNs) were slightly greater (GL261, ~1.60-fold; B16, ~1.44-fold) in the tumor-bearing groups than those in the groups without tumors and with intact MLVs (Fig. 2c). In contrast, no differences in CLN volume were found between the tumor-bearing and tumor-free groups in mice with defective MLVs (Fig. 2c). These results suggest that meningeal lymphatics are required for CLN responses to brain tumors. To directly demonstrate that MLVs→CLNs is a functional path draining tumor fluid, we injected 70-kDa fluorescein isothiocyanate (FITC)-dextran into melanomas or gliomas implanted in the striatum and found a significant amount of FITC-dextran accumulation in the CLNs, which was reduced in the CLNs of mice with defective dorsal MLVs (Fig. 2d). These data indicate that the drainage of macromolecules from intracranial tumors to the CLNs is dependent on intact and functional dorsal MLVs. We found no significant differences of LYVE-1^+^ area in dCLNs between MLV-intact mice and MLV-defective mice without tumor injection (Fig. 2d). In addition, MLV-intact tumor-bearing mice exhibited strong lymphangiogenesis in dCLNs, but dCLNs from MLV-defective mice showed little response to tumor (Fig. 2d). These data suggested that the tumor-induced lymphangiogenesis of sentinel LN is dependent on MLV.

In recent years, increasing evidence has shown that patients with malignant cerebral tumors develop extracranial metastases, especially after a craniotomy or cranial irradiation, and this usually worsens the prognosis.^34–36^ Given the important roles of LVs in metastasis, we hypothesized that dorsal MLVs contribute to the dissemination of brain tumor cells. To test this, GL261-GFP^+^ or B16-GFP^+^ tumor cells were injected into the cisterna magna (i.c.m.) of WT mice. One week later, we found that GFP^+^ tumor cells overlapped with LYVE-1^+^ MLV structures (Supplementary information, Fig. S5a), suggesting that the GFP^+^ cells invade MLVs. This was confirmed by confocal microscopic analysis using orthogonal sectioning with horizontal and vertical views (Supplementary information, Fig. S5b) and 3-D projection analysis using a confocal program at different angles (Supplementary information, Fig. S5c, d). Although the lymphatic invasion events were observed in basal MLVs, the number of invaded tumor cells was significantly lower than that in dorsal MLVs in both GL261 and B16 models (Supplementary information, Fig. S5e–g). The lymphatic invasion events were hardly detected in nasal LVs (Supplementary information, Fig. S5g). These findings suggest that MLVs serve as the conduit for tumor cell spread. To determine whether tumor cells use dorsal MLVs as routes to further spread to CLNs, GL261-GFP^+^ or B16-GFP^+^ cells were i.c.m. injected into mice with ablated dorsal MLVs (Visudyne + Laser) and control mice (Vehicle + Laser). Two weeks later, we harvested CLNs for histology, and calculated the frequency of GFP^+^CLN for metastasis quantification.^37^ In control groups with intact MLVs, we found that few mice developed dCLN metastasis (5/30, 16.7%) in the GL261 tumor model, but most mice developed dCLN metastasis in the B16 tumor model (25/30, 83.3%). However, ablation of the dorsal MLVs significantly inhibited dCLN metastasis (GL261, 1/30, 3.3%; B16, 7/30, 23.3%) (Fig. 2e). Although rare in tumor-bearing control mice (GL261, 1/30, 3.3%; B16, 9/30, 30.0%), sCLN metastasis was barely detectable in dorsal MLV-defective mice (GL261, 0/30, 0%; B16, 2/30, 6.7%) (Fig. 2e). These findings suggest that sprouting and dilation of the dorsal MLVs may facilitate tumor cell entry and spread to CLNs.

### Dorsal MLVs are required for DC trafficking to dCLNs

**Fig. 3:**
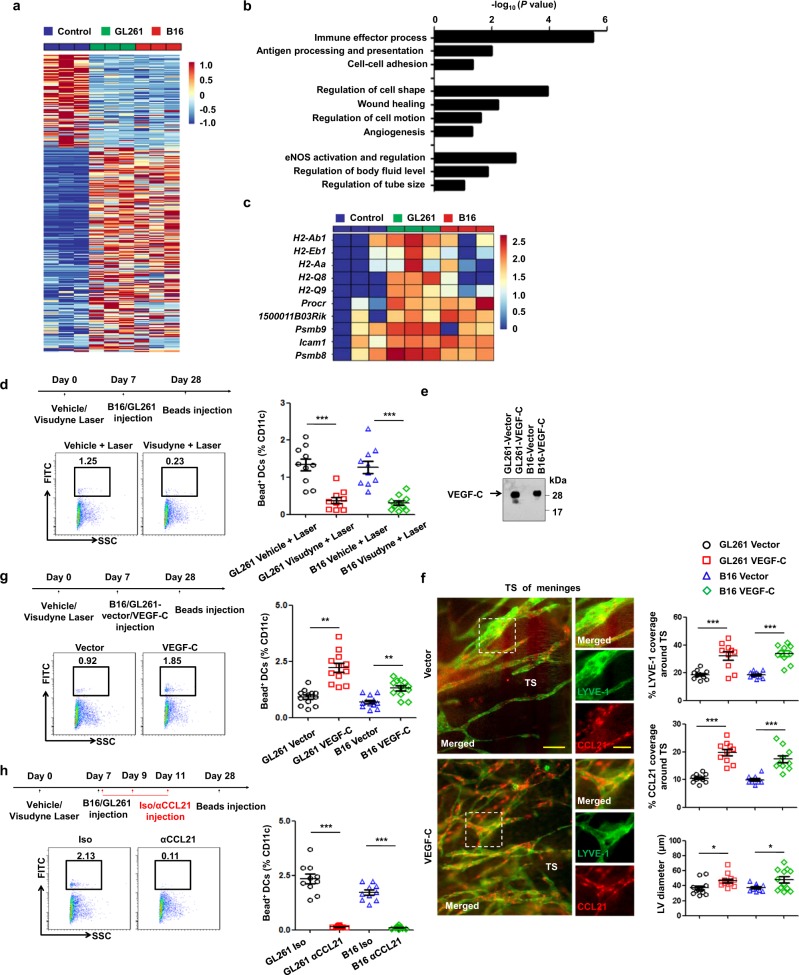
Dorsal MLVs are the main route for immune cell entry to draining CLNs. **a** Heat map of DEGs (Up, 219; Down, 100; power > 0.4). **b**, **c** Gene sets involved in lymphatic remodeling, fluid drainage, as well as inflammatory and immunological responses as shown by the representative upregulated pathways in GL261 tumor-associated and B16 tumor-associated MLECs compared to control MLECs (**b**), and heat map of DEGs enriched in the antigen processing and presentation pathway (**c**). **d** Left panels, treatment scheme and representative flow cytometry dot plots of DC trafficking from GL261 tumors to dCLNs in mice treated with Vehicle + Laser or Visudyne + Laser, determined by the quantity of CD11c^+^MHCII^+^FITC^+^ cells in the dCLNs 24 h after intratumoral injection of FITC-labeled latex beads. Right panel, quantification of Bead^+^ DCs in the dCLNs of mice treated with Vehicle + Laser or Visudyne + Laser. **e** Immunoprecipitation of secreted VEGF-C protein (arrow) in conditioned medium from GL261-Vector, GL261-VEGF-C, B16-Vector, and B16-VEGF-C cells. **f** Left panels, LYVE-1 and CCL21 staining of MLVs in mice bearing Empty and VEGF-C-overexpressing GL261 tumors in the striatum (scale bars, 100 µm in wide-fields; 50 µm in insets). Right panels, quantification of the percentage area of LYVE-1 and CCL21 (*n* = 10). **g** Left panels, treatment scheme and representative flow cytometry dot plots of DC trafficking in the dCLNs of mice bearing GL261 tumors overexpressing Vector or VEGF-C. Right panel, quantification of bead^+^ DCs in dCLNs (*n* = 10). **h** Left panels, treatment scheme and representative flow cytometry dot plots of DC trafficking in the dCLNs of GL261 tumor-bearing mice treated with CCL21 (αCCL21)- or IgG (Iso)-blocking antibodies. Right panel, quantification of bead^+^ DCs in dCLNs (*n* = 10). Data are presented as means ± SEM. *P < 0.05, **P < 0.01, ***P < 0.001; two-way ANOVA (**d**, **f–h**). Data are from at least two (**a**–**c**) or three (**d**–**h**) independent experiments.

To better understand the mechanism underlying the remodeling of dorsal MLVs and gain insights into their involvement in regulating the intracranial tumor environment, we analyzed the transcriptomic profile of MLECs responding to brain tumors. First, 2 weeks after subdural injection of GL261 cells, B16 cells, or PBS as control into mice, dorsal meninges were carefully excised and digested for MLEC sorting, and RNA-seq was performed to investigate the transcriptomic characteristics of tumor-associated MLECs (Supplementary information, Table S1). The MLECs were sorted by gating with a CD45^−^CD31^+^PDPN^+^ strategy (Supplementary information, Fig. S6a), as previously reported.^8^ Principal component analysis revealed distinct clusters of control and tumor-associated MLECs (Supplementary information, Fig. S6b). The populations in each group generally expressed genes that encode classical LEC markers, with no significant differences (Supplementary information, Fig. S6c). We next investigated the differences in their transcriptional profiles. Compared to control MLECs, 219 genes were upregulated and 100 downregulated (power > 0.4) in tumor-associated MLECs by differential expression analysis (Supplementary information, Table S2; Fig. 3a). Gene Ontology analysis showed functional alterations in the MLECs responding to brain tumors. There were changes in several pathways associated with MLV remodeling and lymphatic flow regulation, such as regulation of cell shape, angiogenesis, eNOS activation and regulation, and regulation of body fluid level (Fig. 3b). Clearly, several pathways associated with the immune response were significantly activated in tumor-associated MLECs, such as immune effector process and antigen processing and presentation (Fig. 3b). Of note, gene sets enriched in antigen processing and presentation pathway consisted of some genes implicated in LEC-immune cell interaction^38,39^ (Fig. 3c). This profile strongly suggests a potentially important role of MLVs in the generation of immune responses against brain tumors. It is known that local LVs are critical for DC trafficking from tumor tissues to draining LNs and the activation of immune responses^40^; thus we assumed that dorsal MLVs would assist in DC trafficking from brain tumors to CLNs. To test this, we quantified DC trafficking after dorsal MLV ablation by intratumoral injection of 0.5 μm FITC-labeled beads that are too large to flow into LVs and instead must be taken up by DCs in the tumor before being transported to dCLNs.^41^ As expected, we found that CD11c^+^MHCII^+^FITC^+^ cells in the dCLNs were dramatically reduced in dorsal MLV-defective mice (Supplementary information, Fig. S7a; Fig. 3d), suggesting that dorsal MLVs are critical for the trafficking of DCs to dCLNs.

As the absence of MLVs impaired DC trafficking to dCLNs, we hypothesized that enhanced meningeal lymphangiogenesis would promote this process. Here, we established stable lines of GL261 and B16 cells overexpressing Vector and VEGF-C. VEGF-C protein secreted into the conditioned medium was enriched by immunoprecipitation and further assessed by western blot analysis (Fig. 3e). Then we injected these cells into the striatum of WT mice. Notably, dorsal MLV density and size were significantly higher in the VEGF-C group than in the Vector group (Fig. 3f), suggesting that VEGF-C promotes tumor-associated meningeal lymphangiogenesis. We then quantified the DC trafficking in the Vector and VEGF-C groups. As expected, DC trafficking was markedly greater in the VEGF-C group than in the Vector group (Fig. 3g). These data suggest that VEGF-C-induced lymphangiogenesis enhances DC trafficking to dCLNs. Because MLEC-derived CCL21 was significantly increased along with enhanced meningeal lymphangiogenesis by VEGF-C (Fig. 3f), we further administered CCL21-blocking antibody to the group overexpressing VEGF-C in order to determine whether CCL21 mediates the process. After CCL21 blockade, a significant reduction in FITC-labeled DCs in the dCLNs was observed in both GL261 and B16 tumors overexpressing VEGF-C (Fig. 3h). Taken together, these results suggest that dorsal MLVs mediate the DC trafficking from brain tumors to dCLNs at least in part via the VEGF-C-CCL21/CCR7 signaling pathway.

### Ablation of dorsal MLVs impairs the efficacy of anti-PD-1/CTLA-4 against intracranial tumors

**Fig. 4:**
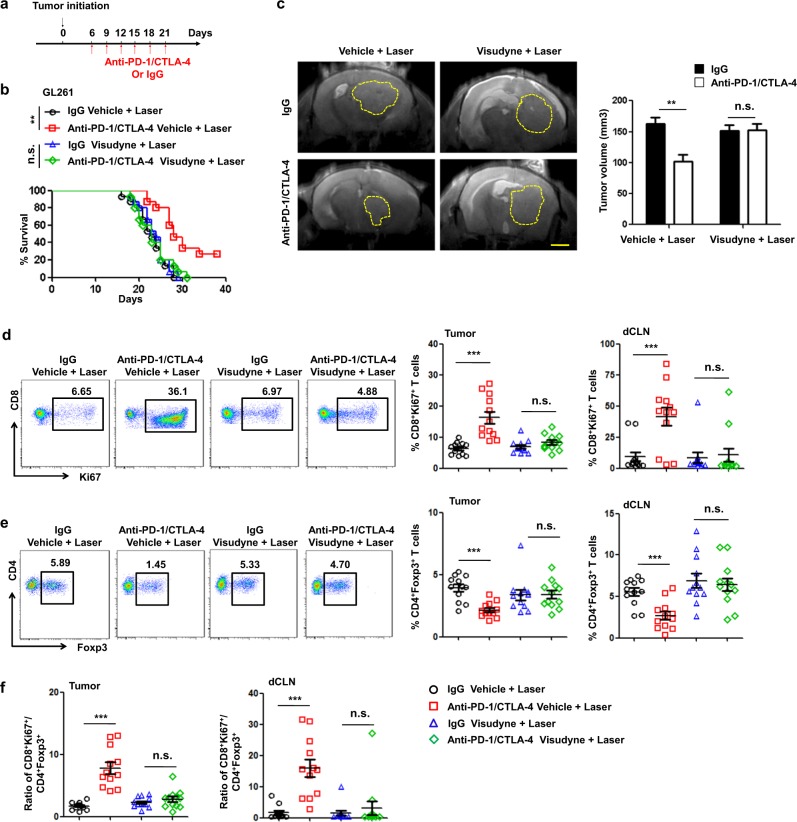
Ablation of dorsal MLVs inhibits anti-brain tumor immune responses. **a** Monitoring and treatment scheme of the administration of anti-PD-1/CTLA-4 or IgG controls (days 8, 9, 12, 16, 18, and 21). **b** Survival of mice treated with Vehicle + Laser or Visudyne + Laser and striatal GL261 tumor injection following the administration of anti-PD-1/CTLA-4 or IgG antibodies (*n* = 15). **c** Representative T2-weighted single brain slices (left panels) and quantification (right panel) of tumor volume in MLV-intact and MLV-defective mice bearing striatal GL261 tumors (*n* = 8). Dashed lines indicate tumor margin. Scale bar, 3 mm. **d** Representative flow cytometry plots of CD8^+^Ki67^+^ T cells in dCLNs (left) and quantification (right) in tumors and dCLNs as percentages of overall CD45^+^ cells on day 14 after inoculation (*n* = 12). **e** Representative flow cytometry plots of CD4^+^Foxp3^+^ T cells in dCLNs (left) and quantification (right) in tumors and dCLNs as percentages of overall CD45^+^cells on day 14 after inoculation (*n* = 12). **f** Ratios of CD8^+^Ki67^+^ T cells to CD4^+^Foxp3^+^ Tregs in tumors and dCLNs. Data are presented as means ± SEM. **P < 0.01, ***P < 0.001, n.s. not significant; long-rank (Mantel–Cox) test (**b**); two-way ANOVA (**c**-**f**). Data are from at least three (**a**, **b**, **d**–**f**) or two (**c**) independent experiments.

Since the lymphatic transport of fluid, antigens, and DCs is critical for activation of the immune response,^42^ we predicted that ablation of dorsal MLVs would change immune cell infiltration in intracranial tumors. As expected, the numbers of CD45^+^, CD68^+^, and CD3e^+^ cells were dramatically reduced both in GL261 and B16 tumors after dorsal MLVs were disrupted (Supplementary information, Fig. S8). These results led us to investigate the role of MLVs in immunotherapy against intracranial tumors. We initiated anti-PD-1/anti-CTLA-4 combination treatment on days 6, 9, 12, 15, 18, and 21 after injection of GL261 cells or B16 cells (Fig. 4a). It has been reported that anti-PD-1 immunotherapy activates T cells in tumor-draining LNs, which contribute to the antitumor response.^29^ We further investigated the impact of CLN resection on the antitumor efficacy of anti-PD-1/anti-CTLA-4 therapy. In control mice, this combination treatment significantly prolonged survival in our model compared to mice receiving IgG antibody. However, the absence of CLNs significantly reduced the treatment efficacy against GL261 tumors and B16 tumors (Supplementary information, Fig. S9a, b). These results suggest that CLNs are necessary in brain tumor immunotherapy.

As described above, DC trafficking into CLNs was impaired when dorsal MLVs were interrupted, therefore we predicted that the anti-PD-1/CTLA-4 combination treatment against brain tumors would have poor effects in MLV-defective mice. Indeed, the combination therapy significantly prolonged survival only in tumor-bearing MLV-intact mice but not in MLV-defective mice (Fig. 4b; Supplementary information, Fig. S9c). Consistent with the survival data, tumor volume, as measured by MRI and GFP, as well as tumor weight significantly decreased in MLV-intact mice after anti-PD-1/CTLA-4 treatment (Fig. 4c; Supplementary information, Fig. S10a–c). As previously reported, this combination therapy activates T cells in both tumor microenvironment and draining LNs, which contribute to the antitumor response.^29,43^ To evaluate the immunological response in brain to this combination therapy, we isolated intracranial tumors and CLNs from GL261-bearing mice, and analyzed the tumor-infiltrating immune cells by flow cytometry as previously reported^28^ (Supplementary information, Fig. S7b, c). Surprisingly, flow cytometry analysis revealed that the therapy strongly increased the total number of CD8^+^ T cells and CD8^+^ Ki67^+^ T cells, and decreased the number of CD4^+^Foxp3^+^ Tregs in both tumors and dCLNs, which reached statistical significance in MLV-intact mice (Fig. 4d–f; Supplementary information, Fig. S11a). Beyond that, the rate of interferon-gamma (IFN-γ)-producing CD8^+^ T cells was significantly increased in MLV-intact mice but not in MLV-defective mice after anti-PD-1/CTLA-4 treatment (Supplementary information, Figs. S7d and S11b). Taken together, these results suggest that dorsal MLVs are critical in immunotherapy against intracranial tumors.

### Enhancement of immunotherapy by VEGF-C is dependent on CCL21/CCR7 signaling

**Fig. 5:**
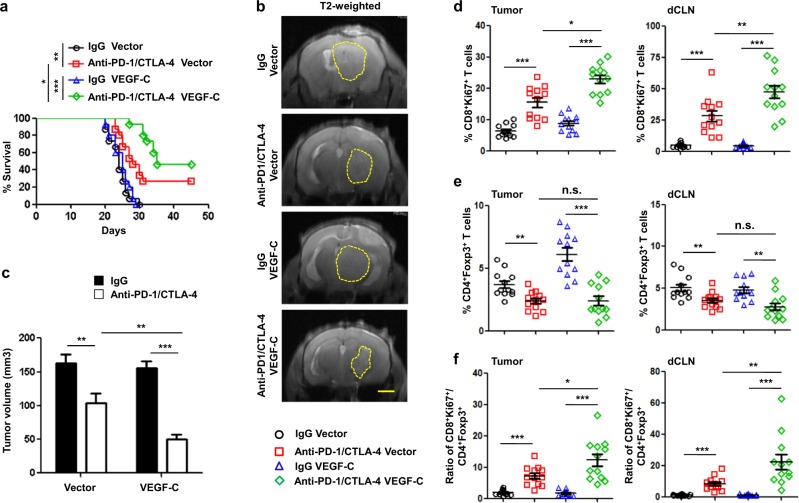
High level of tumor-derived VEGF-C improves anti-PD-1/CTLA-4 efficacy. **a** Survival of mice with striatal Vector- or VEGF-C-overexpressing GL2161 tumors following the administration of anti-PD-1/CTLA-4 or IgG controls (*n* = 15). **b** Representative T2-weighted single brain slices from mice with intracranial injection of GL261 cells overexpressing Vector or VEGF-C (*n* = 6). Scale bar, 3 mm. **c** Tumor volumes in mice with striatal injection of GL261 cells overexpressing Vector or VEGF-C (*n* = 6). **d, e** Quantification of CD8^+^Ki67^+^ T cells (**d**) and CD4^+^Foxp3^+^ T cells (**e**) as percentages of overall CD45^+^ cells in tumors and in dCLNs on day 14 after inoculation (*n* = 12 in each). **f** Ratios of CD8^+^Ki67^+^ T cells to Tregs in tumors and in dCLNs. Data are presented as means ± SEM. *P < 0.05, **P < 0.01, ***P < 0.001, n.s. not significant; long-rank (Mantel–Cox) test (**a**); two-way ANOVA (**c**–**f**). Data are from at least three (**a**, **d**–**f**) or two (**b**, **c**) independent experiments.

Given the importance of the meningeal lymphatic vasculature for successful immunotherapy, we hypothesized that enhanced meningeal lymphangiogenesis would potentiate the susceptibility to immunotherapy. Accordingly, we found that, following the administration of anti-PD-1/CTLA-4, mice bearing tumors overexpressing VEGF-C showed longer survival than mice bearing control tumors (Fig. 5a). Consistently, mice bearing tumors overexpressing VEGF-C displayed decreased tumor volumes and tumor weight compared to Vector group (Fig. 5b, c; Supplementary information, Fig. S10d–f). Notably, these beneficial effects of VEGF-C overexpression was abolished in MLV-defective mice (Supplementary information, Fig. S12), suggesting the importance of intact MLV structure. Next, we investigated the mechanisms underlying the treatment effects by assessing immune cell subsets in brain tumors and dCLNs. We found that anti-PD-1/CTLA-4 treatment increased the CD8/Treg ratios within tumors and dCLNs in both the VEGF-C and Vector groups. However, the CD8/Treg ratio was significantly higher in the VEGF-C than in the Vector groups (Fig. 5d–f). The stronger immune response may be closely associated with the enhanced DC trafficking in the group overexpressing VEGF-C compared to the Vector group (Fig. 3g). These results suggest that increased DC trafficking to dCLNs under enhanced meningeal lymphangiogenesis elicits stronger immune responses.

**Fig. 6:**
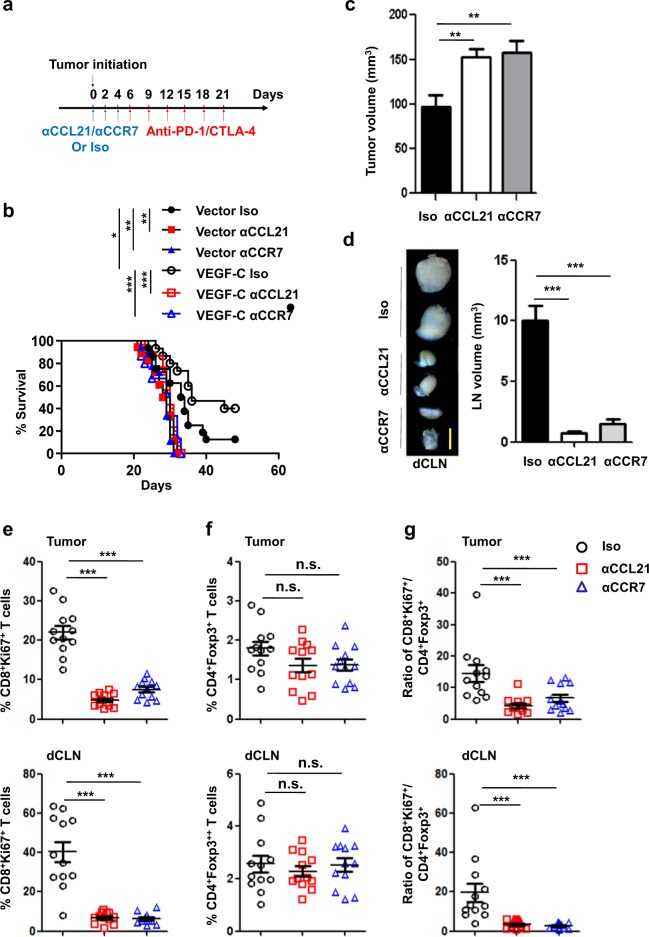
Enhancement of immunotherapy by VEGF-C is dependent on CCL21/CCR7 signaling. **a** Monitoring and treatment scheme. CCL21/CCR7 were blocked on days 0, 2, and 4 before the administration of anti-PD-1/CTLA-4 antibodies. **b** Survival of mice with striatal Vector- or VEGF-C-overexpressing GL2161 tumors following the administration of anti-CCL21, anti-CCR7, or IgG (Iso) antibodies combined with anti-PD-1/CTLA-4 antibodies (*n* = 15). **c** Tumor volumes in mice with striatal injection of GL261 cells overexpressing VEGF-C following the administration of anti-CCL21, anti-CCR7, or IgG (Iso) antibodies combined with anti-PD-1/CTLA-4 antibodies (*n* = 8). **d** Examples (left) and quantification (right) of dCLN volume after GL261 cell injection followed by the administration of anti-CCL21, anti-CCR7, or IgG (Iso) antibodies combined with anti-PD-1/CTLA-4 antibodies (*n* = 12). Scale bar, 1 mm. **e, f** Quantification of CD8^+^Ki67^+^ T cells (**e**) and CD4^+^Foxp3^+^ T cells (**f**) as percentages of overall CD45^+^ cells in tumors and in dCLNs on day 14 after inoculation (*n* = 12 for each). **g** Ratios of CD8^+^Ki67^+^ T cells to Tregs in tumors and in dCLNs. Data are presented as means ± SEM. *P < 0.05, **P < 0.01, ***P < 0.001, n.s. not significant; long-rank (Mantel–Cox) test (**b**); two-way ANOVA (**c**–**g**). Data are from at least two independent experiments (**a–g**).

Next, we wanted to know whether the CCL21/CCR7 pathway mediates the facilitation of antitumor immunotherapeutic effects induced by VEGF-C overexpression. To test this, isotype IgG control or anti-CCL21 and anti-CCR7 antibodies were each administered to mice bearing GL261 control tumors or tumors overexpressing VEGF-C, followed by anti-PD-1/CTLA-4 treatment a few days later to avoid cofounding effects (Fig. 6a). Under these conditions, anti-CCL21 and anti-CCR7 antibodies abolished survival benefit of anti-PD-1/CTLA-4 for mice with GL261 tumors overexpressing VEGF-C (Fig. 6b). Consistently, the tumor volumes were significantly larger in the anti-CCL21 and anti-CCR7 treatment groups than in the IgG control group (Fig. 6c). Interestingly, the swelling of dCLNs was weakened after anti-CCL21 or anti-CCR7 treatment in tumor-bearing mice (Fig. 6d). We further analyzed the lymphocyte changes within tumors and dCLNs from these mice. CD8^+^Ki67^+^ T cells were dramatically decreased within tumors and dCLNs after anti-CCL21 or anti-CCR7 treatment (Fig. 6e). Although CD4^+^Foxp3^+^ Tregs showed no significant differences from mice treated with IgG antibodies (Fig. 6f), the ratios of CD8^+^Ki67^+^ T cells/CD4^+^Foxp3^+^ Tregs significantly differed between the antibody-blocking and control groups (Fig. 6g). Collectively, CCL21 or CCR7 blockade reversed the increased efficacy in mice bearing tumors overexpressing VEGF-C in response to anti-PD-1/CTLA-4 therapy, suggesting that VEGF-C may potentiate checkpoint therapy and this is dependent on the CCL21/CCR7 pathway.

## Discussion

MLVs have been linked to neuroinflammation^10^ and age-associated diseases such as Alzheimer’s disease,^13^ but their role in brain tumor pathology is unclear. Here we demonstrated that MLVs meet three critical criteria to constitute a critical part of the drainage system for brain tumors: (1) they are required for draining interstitial fluid components from melanomas or gliomas implanted in the striatum into CLNs (Fig. 2d); (2) they serve as a lymphatic conduit for tumor cell dissemination from CSF into CLNs (Fig. 2e); and (3) they enable DC trafficking from tumors implanted in the striatum to CLNs (Fig. 3d). Such a draining role was further supported by the data from loss-of-function and gain-of-function experiments: (1) ablation of meningeal lymphatics impaired the drainage of tumor fluid, tumor cells, and DCs from the CNS to draining LNs (Fig. 3d); and (2) enhanced MLV remodeling increased DC trafficking from brain tumors to CLNs (Fig. 3g). So far, several lymphatic routes have been demonstrated in the fluid drainage from the CNS, such as nasal LVs,^44^ basal and dorsal MLVs.^8,11^ However, the main route(s) for macromolecule clearance from the CSF to the lymphatic system has been controversial. Functionally, occlusion of the nasal LVs increases intracranial pressure,^45^ ablation of dorsal MLVs aggravates Alzheimer’s disease,^13^ and basal MLVs are impaired with aging,^10^ suggesting that all these routes play certain roles in facilitating drainage from the CNS. In our study, extensive lymphatic remodeling was mainly observed around the TS and SSS in the dorsal meninges under brain tumor conditions, while basal MLVs displayed mild remodeling at late stage (Supplementary information, Fig. S2a). Consistently, while lymphatic invasion events were observed in basal MLVs, the number of invaded tumor cells was significantly lower than that in dorsal MLVs (Supplementary information, Fig. S5a–f). Along this line, ablation of dorsal MLVs decreased dCLN metastasis (Fig. 2e). Importantly, specific disruption of dorsal MLVs significantly impaired DC trafficking to dCLNs and the immunotherapeutic efficacy against brain tumors in our mouse models (Figs. 3d and 4b). Together, our findings reveal a significant role of dorsal MLVs in brain tumor drainage and immunity.

It should be emphasized, however, that our results cannot exclude the role of basal MLVs and nasal LVs in brain tumor drainage and immunity. In fact, the role of basal MLVs and nasal LVs in draining of CSF components and immune cells had been described in several studies.^11,14,44^ Along this line, some studies using tracer infusion indicated that basal MLVs are the major pathway for CSF outflow.^14–16^ Furthermore, a very recent report showed a reduction in lymphatic outflow of CSF in GL261 glioma model.^46^ In this study, we also found that basal MLVs underwent remodeling and exhibited tumor cell invasion (Supplementary information, Figs. S2a, S5e, f), although they were relatively weak compared to dorsal MLVs. Currently, due to physical location, it is technically challenging to precisely implant tumor cells close to basal MLVs. In addition, it is difficult to specifically ablate basal MLVs. Therefore, further efforts are needed to evaluate individual roles of basal MLVs and nasal LVs in brain tumor immunity. It should also be noted here that tumor cell invasion of CLNs is frequently reported in patients with brain tumors,^47,48^ indicating that lymphatic routes might be involved in extracranial metastasis. As mentioned above, lymphatic tumor cell invasion events were easily detected in both dorsal and basal MLVs (Supplementary information, Fig. S5a–f), and ablation of dorsal MLVs blocked dCLN metastasis (Fig. 2e), suggesting that dorsal MLVs at least partly contribute to extracranial metastasis, while the clinical significance awaits further investigation.

Consistent with a previous finding that tumor-draining lymphatics are indispensable for checkpoint therapy,^29,40^ we found that CLN-mediated immune activation was essential for anti-PD-1/CTLA-4 efficacy (Supplementary information, Fig. S9a, b). Surgical resection of CLNs significantly decreased the immune infiltration and abolished the tumor regression induced by anti-PD-1/CTLA-4 combination therapy (Supplementary information, Fig. S9a, b). Dorsal MLV ablation decreased the efficacy of anti-PD-1/CTLA-4 combination therapy for GL261 tumors (Fig. 4b), suggesting that the dorsal MLVs are necessary for successful anti-PD-1/CTLA-4 combination therapy, though the role of basal MLVs is still uncertain. Interestingly, MLVs are also required for the successful immunotherapy for intracranial B16 tumors in the combined model (mice bearing both striatal and subcutaneous tumor) because ablation of MLVs impaired efficacy of checkpoint inhibition of intracranial B16 tumors (Supplementary information, Fig. S9c). Mechanistically, the traffic of DCs from brain tumor to CLNs and its antigen presentation may contribute to the activation of CD8^+^ T cell against brain tumor, which was proposed to be enhanced by subcutaneous tumor.^49^ Indeed, the ablation of dorsal MLVs resulted in decreased DC drainage into CLNs in mice bearing intracranial GL261 and B16 tumors (Fig. 3d). As an important lymphangiogenic factor, VEGF-C may play a pivotal role in the potentiation of combination therapy against intracranial tumors: MLV lymphangiogenesis and DC trafficking to CLNs simultaneously increased in mice bearing tumors overexpressing VEGF-C, and they responded better to anti-PD-1/CTLA-4 combination therapy (Figs. 3g and 5a). These beneficial effects were abolished by either dorsal MLV ablation or CCL21/CCR7 blockade (Supplementary information, Fig. S12; Fig. 6b). Notably, VEGF-C increases CCL21 level of MLECs (Fig. 3f). Taken together, these data suggest that VEGF-C potentiates checkpoint therapy via a CCL21/CCR7 pathway. Interestingly, a previous clinical study of metastatic melanoma showed that VEGF-C concentrations were positively correlated with the long-term patient responses to combined checkpoint blockade in the periphery.^28^

In summary, our study demonstrates the roles of dorsal MLVs in intracranial tumor fluid drainage and immunity, and shows that the MLV remodeling induced by brain tumors is critical for immunotherapy, mainly through a VEGF-C/CCL21 signaling pathway. Our work sheds new light on the function of meningeal lymphatic vasculature in anti-brain tumor immunity, suggesting that augmentation of MLV function might be a promising therapeutic approach for enhancing the efficacy of systemic immunotherapy against brain tumors.

## Materials and methods

### Animals and treatments

Male C57BL/6 mice (6–8 weeks old) were used. The *Prox1-CreER*^*T2*^ line (C57BL/6) was a gift from Dr Taija Makinen (Department of Immunology, Genetics and Pathology, Uppsala University, Sweden).^50^ The *Prox1-CreER*^*T2*^ mice were interbred with *R26-tdTomato* mice (C57BL/6, kindly provided by A.H., Peking University, Beijing, China) to generate *Prox1-CreER*^*T2*^*; R26-tdTomato* mice. For lineage tracing, experimental mice were given 100 µL of 10 mg/mL tamoxifen by intraperitoneal (i.p.) injection daily for 4 consecutive days. Three weeks after the last injection, the Cre recombination efficiency was confirmed. Tumor-bearing mice were treated on days 6, 9, 12, 15, 18, and 21 after tumor inoculation by i.p. injection of IgG (200 µg) antibody, or combined PD-1 (200 µg) and CTLA-4 (200 µg) antibodies. For CCL21 or CCR7 blocking assays, tumor-bearing mice were treated on days 0, 2, or 4 by i.p. injection of goat or mouse IgG isotype control antibody (10 µg), CCL21-blocking antibody (10 µg), or CCR7-blocking antibody (10 µg) before anti-PD-1/CTLA-4 combined therapy. Animals were maintained in the Center for Experimental Animals (a facility accredited by the Association for Assessment and Accreditation of Laboratory Animal Care) at Peking University, Beijing, China. All procedures involving animals followed protocols approved by the Committee for Animal Research of Peking University and conformed to the Guide for the Care and Use of Laboratory Animals.

### Reagents and antibodies

Tamoxifen (T5648), Innomycin (I0634), and phorbol 12-myristate 13-acetate (PMA; P1585) were from Sigma; collagenase type I (4196) was from Worthington; and collagenase type II (17101-015) was from Gibco. RIPA buffer (R0020) was from Solarbio Life Science. FITC-conjugated latex microspheres (17152-10) were from Polysciences and Visudyne was from MedChemExpress (HY-B0146). The cell strainers (70 µm, 352350) and Golgi plugs (51-2301KZ) were from BD Bioscience. The following antibodies were used for OCT-embedded sections: anti-LYVE-1 (rabbit, 1:500, Abcam, ab14917), anti-GFP (rabbit, 1:500, Novus Biologicals, NB600-308), anti-Prox1 (goat, 1:100, R&D Systems, AF2727), anti-CD31 (rat, 1:500, BD Bioscience, 553370), anti-CD3e (goat, 1:500, eBioscience) and anti-CCL21 (goat, 1:500, R&D System, AF457). Alexa Fluor 488/555/647 donkey/goat anti-rabbit/rat/goat IgG antibodies were from Invitrogen. Anti-VEGF-C antibody (mouse, 1:500, Santa Cruz, sc-374628) was used for western blot analysis. The following antibodies were used for fluorescence-activated cell sorting (FACS): anti-CD31-PE-Cy7 (1:100, eBioscience, 25-0311-82), anti-LYVE-1-eFluor 660 (1:100, eBioscience, 50-0443-82), anti-PDPN-PE (1:100, eBioscience, 12-5381-82), anti-CD45-FITC (1:100, BD Bioscience, 553079), anti-CD45-Percp-5.5 (1:300, Invitrogen, 45-0451-82), anti-CD3-FITC (1:300, Invitrogen, 11-0032-82), anti-CD4-APC (1:300, Invitrogen, 17-0042-82), anti-CD8a-APC (1:300, Invitrogen, 17-0081-82), anti-CD11C-PE (1:300, Invitrogen, 12-0114-82), anti-MHCII-Super Bright 702 (1:300, Invitrogen, 67-5321-82), anti-Foxp3-PE (1:300, Invitrogen, 12-5773-80), anti-Ki67-PE (1:300, Invitrogen, 12-5698-80), anti-IFN-γ-PE-Cy7 (1:300, Invitrogen, 25-7311-82), rat IgG2b isotype-PE (1:300, Invitrogen, 12-4031-81), rat IgG2b isotype-PE-Cy7 (1:300, Invitrogen, 25-7311-82), and fixable viability stain 510 (1:5000, BD Bioscience, 564406). Anti-PD-1 antibody (BE0146), anti-CTLA-4 antibody (BE0164), mouse IgG isotype control (BE0086), and rat IgG isotype control (BE0089) for animal treatment were from Bioxcell. Anti-CCR7 blocking antibody (16-1971) was from Invitrogen.

### Cell cultures

293T cells were cultured in Dulbecco’s modified Eagle’s medium (DMEM) containing 10% fetal bovine serum (FBS); mouse B16 melanoma cells were maintained in RPMI 1640 medium with 5% FBS; and mouse glioma GL261 cells were maintained in DMEM with 10% FBS. They were all cultured at 37 °C in a humidified atmosphere of 5% CO_2_.

### Establishment of GL261 and B16 cells overexpressing GFP or VEGF-C

We constructed retroviral vectors encoding GFP or VEGF-C. The retrovirus was made in 293T cells, and the culture supernatant was used to infect B16 and GL261 cells. Puromycin (0.75 mg/L) was used to screen for stable cell pools after infection with GFP or VEGF-C.

### Ablation of dorsal MLVs

Mice aged 7–8 weeks were anesthetized by i.p. injection of 1% pentobarbital sodium. Visudyne was reconstituted following the manufacturer’s instructions (2 mg/mL), and 5 µL was injected into the cisterna magna. After 15 min, Visudyne was photoconverted with a nonthermal 689-nm wavelength laser light on different spots through the intact skull as previously reported.^10^

### Animal model with subdural injection

Mice aged 7–8 weeks were anesthetized by i.p. injection of 1% pentobarbital sodium and fixed in a stereotactic frame. After shaving the head, the skull was exposed and a small hole was made on the left side 4 mm anterior to the interaural line and 2.5 mm lateral to the midline. One microliter of PBS containing 50,000 GL261 cells or 10,000 B16 cells was injected subdurally with a 30-gauge Hamilton syringe at a depth of 1 mm, into the space between the dura and brain. One microliter of PBS was injected as control, and then the skin was closed and mice were allowed to recover on a heat pad. Mice injected with tumor cells were maintained for no more than 4 weeks.

### Animal model with striatal injection

Mice aged 7–8 weeks were anesthetized by i.p. injection of 1% pentobarbital sodium and fixed in a stereotactic frame. After shaving the head, an incision was made and the skull was exposed. One microliter of PBS containing 50,000 GL261 cells or 10,000 B16 cells was stereotactically injected at 2 mm lateral to bregma at a depth of 3 mm below the dura with a 30-gauge Hamilton syringe. The injection was performed over a 10-min period. One microliter of PBS was injected as control, the skin was closed, and mice were allowed to recover on a heat pad. In the B16 melanoma models, since an extracranial tumor is required for successful intracranial immunotherapy according to a previous report,^49^ we combined a striatal injection with a subcutaneous injection. For subcutaneous injection, 200,000 B16 cells were injected subcutaneously on the flank. Mice injected with tumor cells were maintained for no more than 6 weeks.

### Animal model of intra-cisterna magna injection

Mice aged 7–8 weeks were anesthetized by i.p. injection of 1% pentobarbital sodium and fixed in a stereotactic frame. The skin of the neck was shaved and cleaned, then an incision was made and the muscle layers were removed to expose the cisterna magna. Using a 30-gauge Hamilton syringe, 5 µL PBS containing 100,000 GL261 or B16 cells was injected into the cisterna magna, and the same volume of PBS was injected as control. After injection, the syringe was left in place for 2 min and removed slowly. The skin was then closed and mice were allowed to recover on a heating pad. Mice injected with tumor cells were maintained for no more than 3 weeks.

### CLN resection

Deep and superficial CLNs were removed from C57 mice as previously described.^51^ Briefly, mice were anaesthetized with 1% pentobarbital sodium and a sagittal incision was made in the middle of the neck. After ligation of the collecting LVs anterior to the dCLN, 8 sCLNs and 2 dCLNs were removed under a surgical microscope. After surgery, buprenorphine (subcutaneous injection) and antibiotic (i.p. injection) were used. Tumor cells were transplanted after 1 week of recovery.

### Quantification of CLN metastasis

B16-GFP^+^ or Gl261-GFP^+^ cells were i.c.m injected into mice with ablated dorsal MLVs (Visudyne + Laser) and control mice (Vehicle + Laser). Two weeks later, dCLNs and sCLNs were harvested as previously reported.^13^ CLNs were fixed in 4% paraformaldehyde (PFA) overnight, followed by dehydration. CLNs were embedded in OCT (Tissue-Tek). The whole CLN was then sectioned for GFP detection. Metastasis was determined as the presence of GFP^+^ tumor cells and quantified by the frequency of GFP^+^ CLN.^37^

### Fluid drainage and DC trafficking

To evaluate the drainage function of MLVs, 10 μL of 0.5% 70-kDa FITC-dextran was injected into the brain tissues or intracranial tumors through a 30-gauge needle. After 10 min, the mice were sacrificed, and the dCLNs and sCLNs were harvested and embedded in OCT. Frozen sections were prepared and fixed in acetone for 10 min at room temperature, and then air-dried and rehydrated in water. They were then permeabilized in 0.3% Triton X-100 in Tris-buffered saline (TBS) and blocked in 10% horse serum. After incubation with anti-LYVE1 antibody diluted in TBS at 4 °C overnight, the sections were washed 3 times with TBS for 5 min each and incubated with fluorophore-conjugated secondary antibody (all 1:500, diluted in TBS) at room temperature for 1 h. The sections were finally washed and incubated with DAPI medium before mounting.

For DC trafficking, 2 μL of 0.5 μm FITC-conjugated latex microspheres were injected into the tumors; 24 h later, LNs were harvested, and single-cell suspensions were prepared and analyzed by flow cytometry for CD11c^+^MHCII^+^DITC^+^DCs.

### Edema analysis

MRI was performed on a 9.4 Tesla MRI scanner (Bruker Biospin, Billerica, MA, USA) as previously described.^52^ Brain edema was assessed by T2 relaxation maps, which were generated from multi-echo spin-echo images. The acquisition parameters were as follows: TE = 10 ms, 10 echoes, TR = 2500 ms, 11 image slices, 0.5 mm slice thickness, 150 mm in-plane resolution, and NA = 2. Voxelwise exponential fitting of the image signal intensity as a function of echo-time was performed (MatLab) to determine T2 relaxation time maps.

### Tumor volume and tumor weight measurement

Tumor volume was measured by MRI and GFP-expressing tumor. Briefly, we used T2-weighted rapid acquisition with relaxation enhancement (RARE) images to assess tumor volume. The acquisition parameters were as follows: TE = 10, RARE factor = 16, TR = 3000 ms, NA = 4, 11 image slices, 0.5-mm slice thickness, and 150 μm in-plane resolution. Tumor area was determined from the T2 hyperintense regions in the brain as previously reported.^52^ Tumor area of GFP-expressing tumor was measured as previously reported.^53^ Briefly, brain was sliced after sacrifice of mice. Brain tumor xenograft was analyzed by bright field and GFP fluorescence imaging. Tumor volume was calculated by length × width^2^ × 0.52.

Tumor weight was measured immediately after tumor was isolated from brain.

### Tissue collection and processing

After anesthesia, mice were transcardially perfused with ice-cold PBS with heparin and then with 4% PFA. The whole brain and skull with meninges were fixed in 4% PFA at 4 °C. Brain tissue and tumors were dehydrated in high concentrations of sucrose at 4 °C, embedded in OCT and stored at −80 °C. The volume of tumors was calculated as 0.52 × length × width^2^. Sections were cut at 5 μm for hematoxylin and eosin staining or immunohistochemistry. The meninges were isolated from the skullcap and subjected to whole-mount staining. The average number of LVs was determined from three microscopic fields with the highest vessel density. The dCLNs were collected from each animal on the day of sacrifice and their volumes were calculated as (π/6) × (length × width)^3/2^.

### Immunostaining

Cryosections of tumors were fixed in acetone for 10 min at room temperature, and then air-dried and rehydrated in water. They were then permeabilized in 0.3% Triton X-100 in TBS and blocked in 10% horse serum. After incubation with primary antibody diluted in TBS at 4 °C overnight, the sections were washed 3 times with TBS for 5 min each and incubated with fluorophore-conjugated secondary antibody (all 1:500, diluted in TBS) at room temperature for 1 h. The sections were finally washed and incubated with DAPI medium before mounting.

For whole-mount staining, the meninges attached to the skull were fixed in 4% PFA, and separated from the skullcap. Then they were incubated with PBS containing 2% horse serum, 1% bovine serum albumin (BSA), 0.1% Triton X-100, and 0.05% Tween 20 for 1 h at room temperature. After incubation with primary antibody diluted in PBS with 1% BSA and 0.5% Triton X-100 at 4 °C overnight, they were washed 3 times with PBS for 5 min each and incubated with fluorophore-conjugated secondary antibody (all 1:500, diluted in PBS with 1% BSA and 0.5% Triton X-100) at room temperature for 1 h. Finally, the meninges were incubated with DAPI medium before capturing images.

Nasal LVs were detected as previously described.^44^ Briefly, the whole heads were fixed in 4% PFA overnight, followed by decalcification in 14% EDTA for 7 days. During decalcification, EDTA was replaced with fresh 14% EDTA every day. The whole heads were dehydrated in 30% sucrose for 3 days and then embedded in Tissue-Tek OCT compound. 60 μm sections were prepared for immunostaining. The protocol for immunostaining of nasal LVs was the same as that of tumor tissue.

### Isolation of MLECs and flow cytometry

For MLEC collection, PBS, GL261 cells, or B16 cells were injected subdurally into mice, which were sacrificed 2–3 weeks later. Briefly, isolated dorsal meninges were incubated in Ca^2+^-PBS containing 0.2% type I and 0.2% type II collagenase at 37 °C for 45 min (*n* = 3 biological replicates per group, each pooled from 6 individual mice). At the end of incubation, the tissue fragments were passed through 70-μm nylon mesh cell strainers after neutralization with 10% FBS in DMEM. The cells were then centrifuged at 2000 rpm at 4 °C for 10 min, the supernatant was removed, and the cells were re-suspended in ice-cold FACS solution (pH 7.4, 0.1 M PBS, 1 mM EDTA, 1% BSA). Further, cells were stained with CD31-PE-CY7, CD45-FITC, LYVE-1-eFluor 660, and fixable viability stain 510. After being washed twice with PBS containing 1% BSA, cells were analyzed and sorted by flow cytometry (BD FACSAria SORP). For RNA-seq, MLECs were collected into PBS containing 1% BSA and kept on ice until lysis and reverse transcription. In experiments requiring analysis of lymphocyte infiltration and activity, intracranial tumors were incubated in Ca^2+^-PBS containing 0.2% type I collagenase at 37 °C for 30 min, and the dCLNs were collected and incubated in PBS containing 0.1% collagenase D at 37 °C for 1 h. At the end of incubation, the tissue fragments were passed through 70-μm nylon mesh cell strainers after neutralization with 10% FBS in DMEM. After centrifugation, the cells were re-suspended and stained with CD45, CD3e, CD4, CD8, Foxp3, Ki67, CD11c, and MHCII. For IFN-γ staining, cells were treated with PMA + Innomycin + Golgi plug for 4 h before staining. Foxp3, Ki67, or IFN-γ staining was performed using the transcription factor Foxp3 staining kit. All FACS analysis was performed with BD LSRFortessa, and gates were set based on isotype-specific control antibodies. All FACS data were analyzed with FlowJo software (Tree Star, Ashland, OR, USA).

### cDNA preparation

The modified RNA-seq followed the previously published studies.^54,55^ Briefly, after FACS purification, MLECs were placed in lysis buffer by mouth pipette. In the reverse transcription reaction, a poly(T) tail was added to the 3′ end of the first-strand cDNAs, which were amplified by 18 cycles for library construction. The amplified cDNAs were fragmented to ~300 bp by Covaris S2, and a KAPA Hyper Prep Kit was used to generate sequence libraries. Paired-end 150-bp sequencing was further performed on an Illumina HiSeq 4000 platform (sequenced by Novogene).

### RNA-seq analysis

First, the Illumina adapter sequences, amplification primer, polyA tail sequences, and reads of low-quality bases (N > 10%) were removed from all the RNA-seq raw data. Then, the processed sequences were aligned to the mm9 mouse reference genome (UCSC) using TopHat (version 2.0.12). Subsequently, the expression level of each gene was calculated using Cufflinks (version 2.2.1). The expression level of each gene was converted to log_2_ (fpkm + 1) for downstream analysis. Principal component analysis was performed using the prcomp function in R to separate different cell groups. We used the Seurat FindAllMarkers function (test.use = “roc”) on normalized FPKM (Fragments Per Kilobase Million) expression values to identify differentially-expressed genes (DEGs). Genes with power > 0.4 were selected. Gene Ontology analysis of DEGs was performed using DAVID (https://david-d.ncifcrf.gov/) (DAVID Bioinformatics Resources 6.7). Heat maps of DEGs and enriched gene sets were created with the R package pheatmap (http://www.broadinstitute.org/gsea/index.jsp). The RNA-seq data have been deposited in Gene Expression Omnibus under accession number GSE128207.

### Immunoprecipitation and western blot

Labeled VEGF-C was immunoprecipitated from conditioned medium using antibodies against Flag, and beads were washed twice in PBS with 0.5% BSA and 0.02% Tween, and once in PBS. VEGF-C immunoprecipitates were then analyzed on SDS-PAGE under reducing conditions, followed by standard western blot analysis.

### Image analysis

The images of meninges were acquired under the 10× lens of a high-content microscope (Molecular Devices, ImageXpress Micro XL). Images of the same region of the TS were acquired under a confocal microscope and a mean of 30 individual LV diameter measurements was calculated for each sample. Confocal images of cryostat sections were acquired under a 40× oil-immersion lens and analyzed by laser-scanning confocal microscopy (Zeiss LSM 510). The number of vessels in the field (4–5 fields per section) of each tumor section (6–8 sections per sample) was counted. The Z-stacks were acquired at 512 × 512 pixel resolution with a z-step using ImageJ software. The images of LNs were acquired under the 10× lens of a fluorescence microscope (Leica), and analyzed using ImageJ software.

### Statistical analysis

All experiments were repeated at least twice. The number of animals is specified in each figure legend. Data are expressed as means ± SEM. Statistical analyses used Student’s *t* test, two-way ANOVA, and the long-rank (Mantel–Cox) test. GraphPad software was used for data analysis. Statistical significance is indicated as follows: *P < 0.05, **P < 0.01, ***P < 0.001, n.s. not significant.

## Supplementary information

Supplementary information, Figure S1

Supplementary information, Figure S2

Supplementary information, Figure S3

Supplementary information, Figure S4

Supplementary information, Figure S5

Supplementary information, Figure S6

Supplementary information, Figure S7

Supplementary information, Figure S8

Supplementary information, Figure S9

Supplementary information, Figure S10

Supplementary information, Figure S11

Supplementary information, Figure S12

Supplementary information, Table S1

Supplementary information, Table S2
