# Supplementary material for: Meningeal lymphatic vessels regulate brain tumor drainage and immunity
Source: Cell Res. 2020 Feb 24;30(3):229–43. doi: 10.1038/s41422-020-0287-8 (PMC7054407; doi:10.1038/s41422-020-0287-8)
Supplement: Supplementary file 1 — Supplementary information, Figure S1 [file 41422_2020_287_MOESM1_ESM.pdf]

Supplementary information, Figure S1

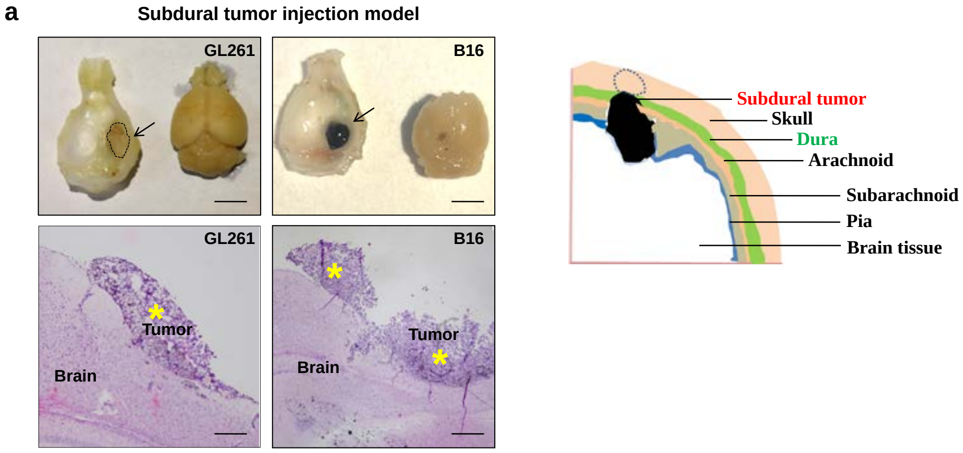

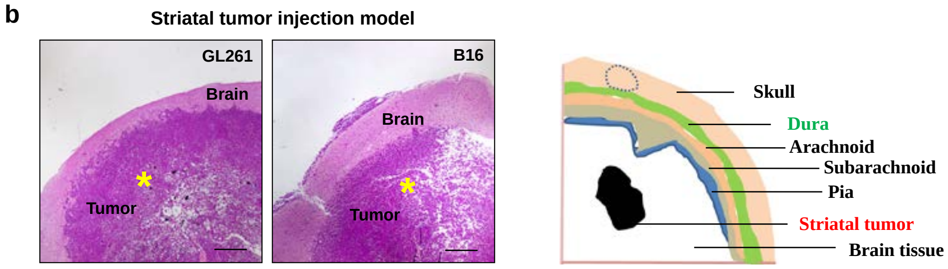

Fig. S1 Anatomy of skull and brain parenchyma in subdural and striatal tumor injection models. a, Upper panels, typical images of skull and brain after subdural injection of B16 cells at day 14. Note that the tumor mass (arrows) grew near the site of injection without invading the surface of the brain, and then typically adhered to the dura when skull and brain were separated. Scale bars, 5 mm. Lower panels, histology of brain parenchyma in subdural injection models (note that tumor cells were mainly in the subdural space but not in the parenchyma, * indicated tumor tissue). Scale bars, 200 µm. **b**, Histology of brain parenchyma in striatal injection models (note that tumor cells were mainly in the parenchyma, * indicated tumor tissue). Scale bars, 500 µm.
