# Supplementary material for: Meningeal lymphatic vessels regulate brain tumor drainage and immunity
Source: Cell Res. 2020 Feb 24;30(3):229–43. doi: 10.1038/s41422-020-0287-8 (PMC7054407; doi:10.1038/s41422-020-0287-8)
Supplement: Supplementary file 2 — Supplementary information, Figure S2 [file 41422_2020_287_MOESM2_ESM.pdf]

Supplementary information, Figure S2

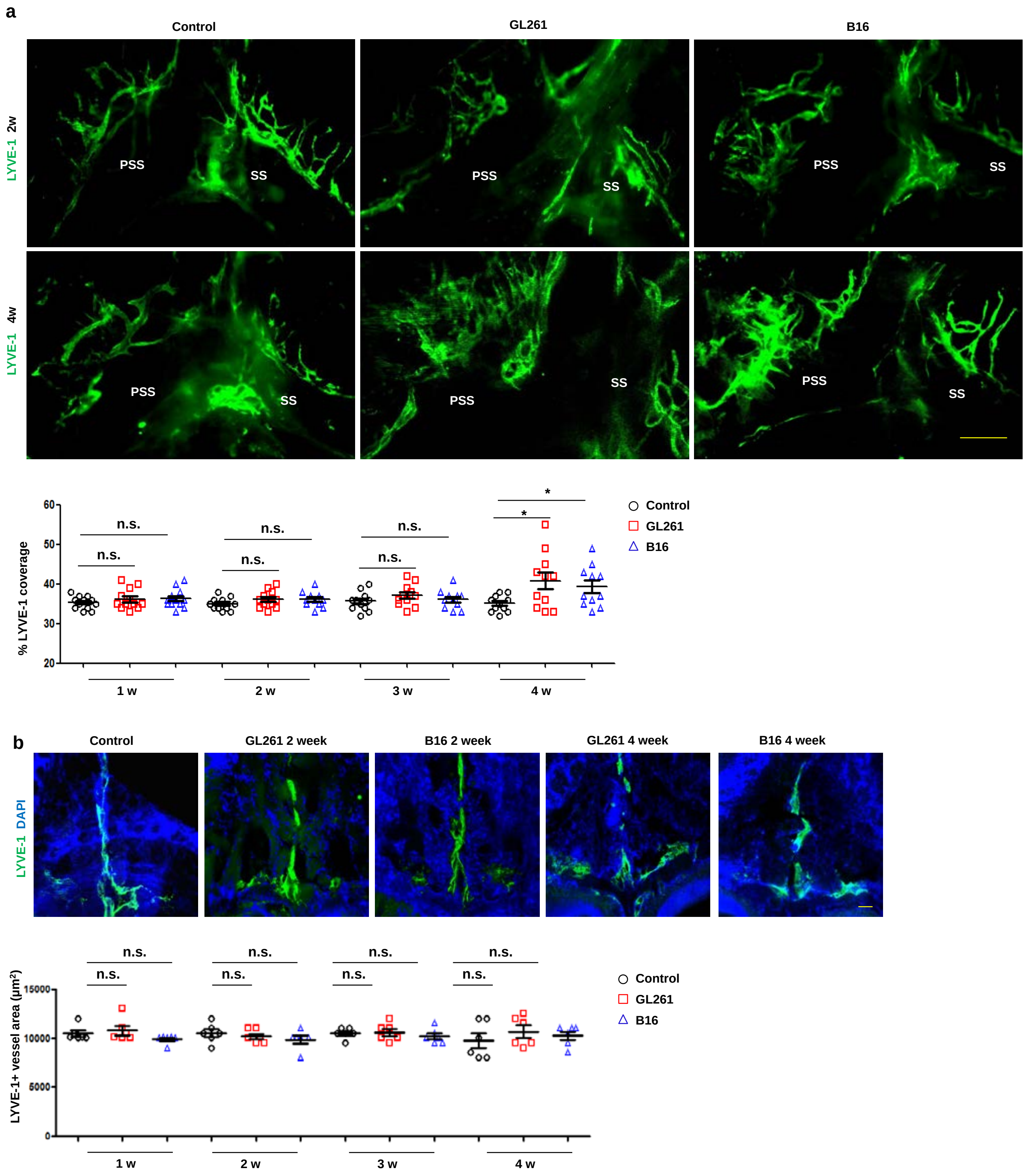

Fig. S2 Brain tumors scarcely induce basal meningeal lymphangiogenesis and nasal lymphangiogenesis. a, Basal meningeal LYVE-1 staining (upper panels) and quantification of LVs around the PSS and SS (lower panels) 1, 2, 3 and 4 weeks after striatal injection of GL261 or B16 cells into WT mice. PSS, petrosquamosal sinus; SS, sigmoid sinus. Scale bar, 500 μm. **b**, Nasal lymphatic LYVE-1 staining (left panels) and quantification (right panel) 1, 2, 3, 4 weeks after striatal injection of GL261 or B16 cells into WT mice. Scale bar, 100 μm. Data are presented as the mean ± SEM. *P <0.05, n.s. not significant; two-way ANOVA (**a**, **b**). Data are from at least three (**a**, **b**) independent experiments.
