# Supplementary material for: Meningeal lymphatic vessels regulate brain tumor drainage and immunity
Source: Cell Res. 2020 Feb 24;30(3):229–43. doi: 10.1038/s41422-020-0287-8 (PMC7054407; doi:10.1038/s41422-020-0287-8)
Supplement: Supplementary file 3 — Supplementary information, Figure S3 [file 41422_2020_287_MOESM3_ESM.pdf]

Supplementary information, Figure S3

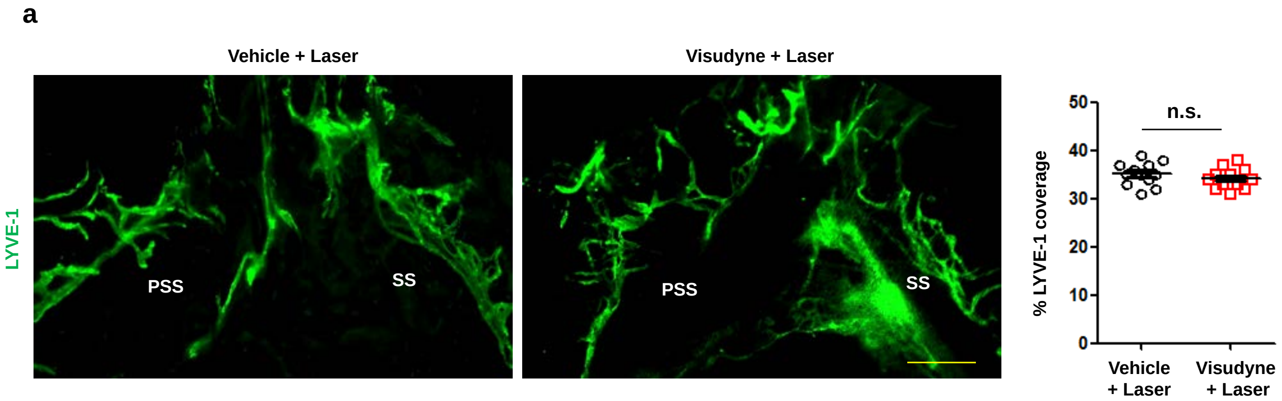

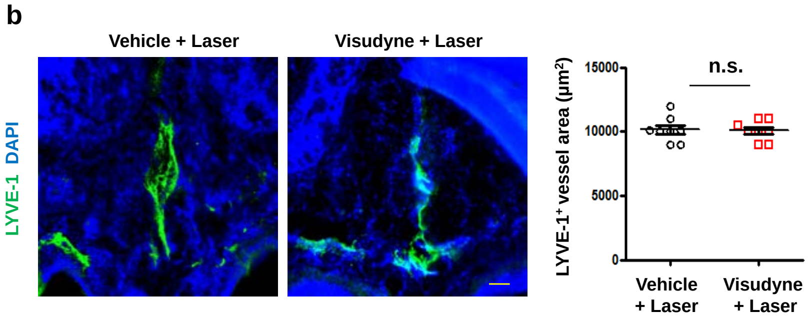

Fig. S3 Ablation of MLVs does not affect basal and nasal LVs. a, Representative images (left panels) and quantification (right panel) of basal LVs around the PSS and SS in mice treated with Vehicle + Laser or Visudyne + Laser. Scale bar, 500 μm. **b**, Representative images (left) and quantification (right) of nasal LVs in mice treated with Vehicle + Laser or Visudyne + Laser. Scale bars, 100 μm. Data are presented as the mean ± SEM. n.s. not significant; two-tailed unpaired Student's t-test (**a**, **b**). Data are from at least three (**a**, **b**) independent experiments.
