# Supplementary material for: Meningeal lymphatic vessels regulate brain tumor drainage and immunity
Source: Cell Res. 2020 Feb 24;30(3):229–43. doi: 10.1038/s41422-020-0287-8 (PMC7054407; doi:10.1038/s41422-020-0287-8)
Supplement: Supplementary file 4 — Supplementary information, Figure S4 [file 41422_2020_287_MOESM4_ESM.pdf]

Supplementary information, Figure S4

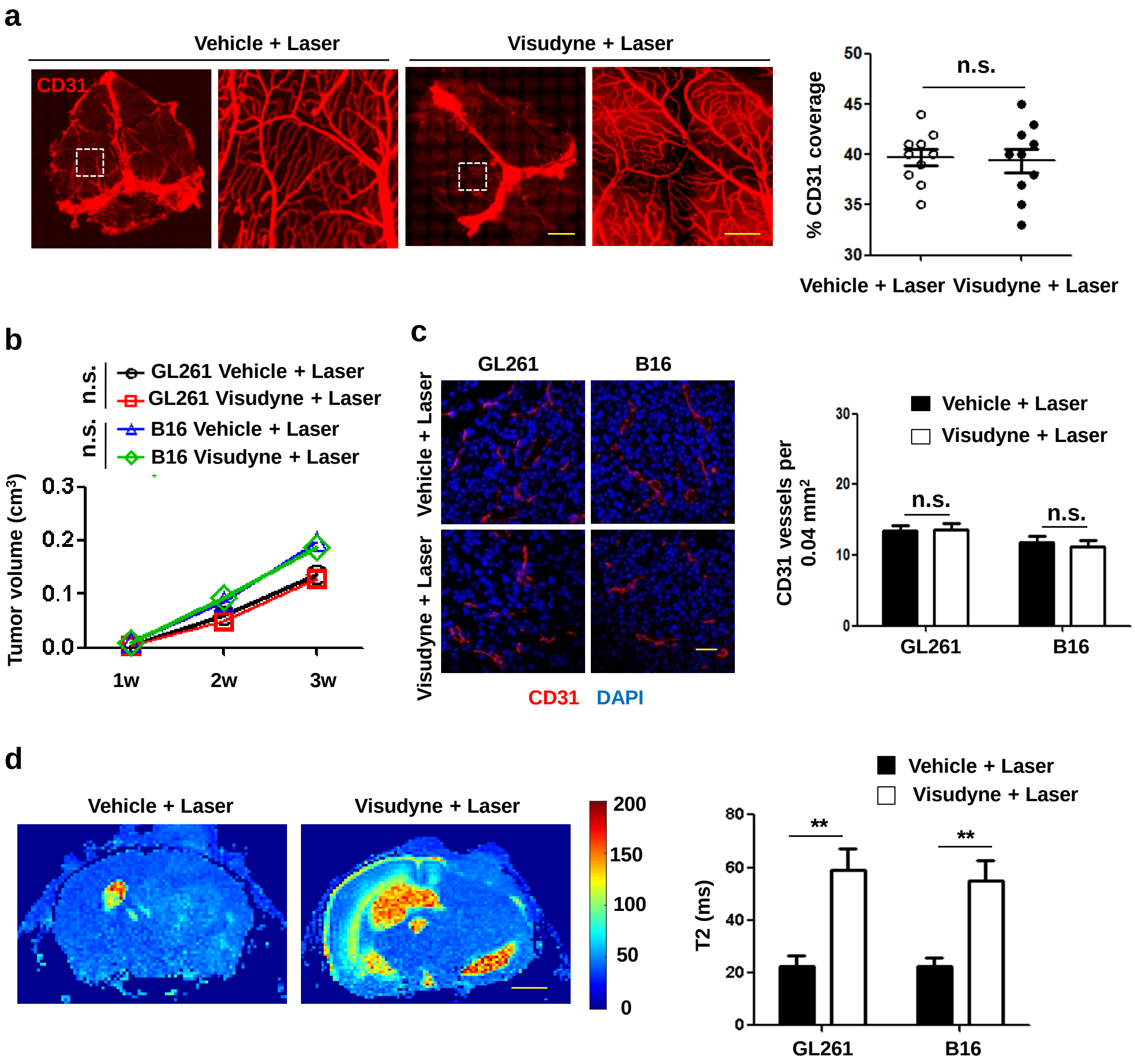

Fig. S4 Ablation of MLVs does not affect meningeal blood vasculature and tumor growth. a, Representative meningeal CD31 staining (left) and quantification of the percentage area of CD31 per meninx (right, *n* = 10). Scale bars, 200 µm in wide-fields; 40 µm in insets. **b**, Tumor growth of GL261 or B16 in mice treated with Vehicle + Laser or Visudyne + Laser in striatal injection models. **c,** Representative images and quantification of CD31 staining in GL261 and B16 subdural tumors from mice treated with Vehicle + Laser or Visudyne + Laser (*n* = 12). Scale bar, 50 µm. **d**, Edema was accessed by the T2 value for brain (T2 value is correlated with water content, *n* = 8). Scale bar, 3 mm. Data are presented as the mean ± SEM. ***P* <0.01, n.s. not significant; two-tailed unpaired Student’s t-test (**a**); two-way ANOVA (**b- d**). Data are from at least three (**a**, **b**) or two (**c, d**) independent experiments.
