# Supplementary material for: Meningeal lymphatic vessels regulate brain tumor drainage and immunity
Source: Cell Res. 2020 Feb 24;30(3):229–43. doi: 10.1038/s41422-020-0287-8 (PMC7054407; doi:10.1038/s41422-020-0287-8)
Supplement: Supplementary file 5 — Supplementary information, Figure S5 [file 41422_2020_287_MOESM5_ESM.pdf]

Supplementary information, Figure S5

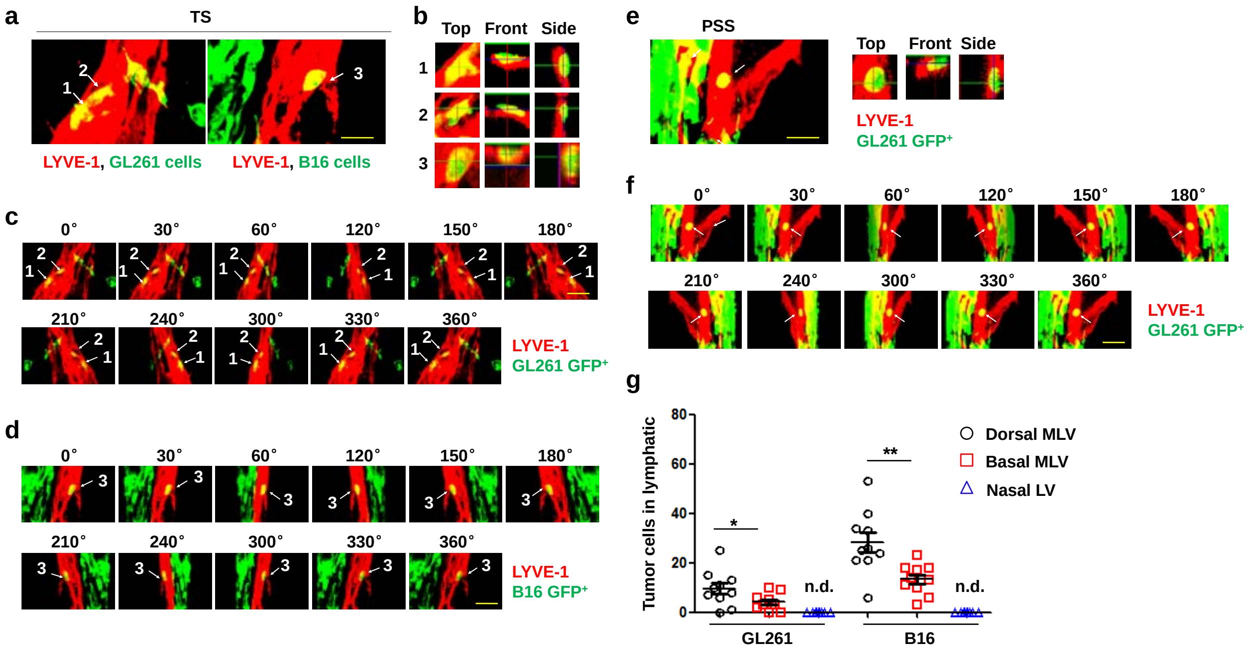

Fig. S5 MLV remodeling facilitate tumor cell invasion. **a**, LYVE-1 staining around the TS 7 days after i.c.m. injection of GL261-GFP$^+$ cells and B16-GFP$^+$ cells into WT mice (arrows, overlapping GFP$^+$ and LYVE-1-positive signals). Scale bar, 20 µm. **b**, Orthogonal sections showing GFP$^+$ cells overlapping with LYVE-1 signals from the top, front, and side. **c**, **d**, Images of GL261-GFP$^+$ (**a**) and B16-GFP$^+$ (**b**) cells (arrows) from different angles (0-360°). Scale bars, 40 µm. **e**, LYVE-1 staining around the PSS 7 days after i.c.m. injection of GL261-GFP$^+$ cells into WT mice (arrows, overlapping GFP$^+$ and LYVE-1-positive signal) and orthogonal sections showing GFP$^+$ cell. Scale bar, 20 µm. **f**, Images of GL261-GFP$^+$ (**e**) from different angles (0-360°). Scale bars, 40 µm. **g**, Quantification of tumor cells invaded into dorsal MLV, basal MLV, and nasal LV per mouse 7 days after i.c.m. injection of GL261-GFP$^+$ cells or B16-GFP$^+$ cells. Data are presented as the mean ± SEM. *P <0.05, **P <0.01; two-way ANOVA (**a-g**). Data are from at least three (**a-g**) independent experiments.
