# Supplementary material for: Meningeal lymphatic vessels regulate brain tumor drainage and immunity
Source: Cell Res. 2020 Feb 24;30(3):229–43. doi: 10.1038/s41422-020-0287-8 (PMC7054407; doi:10.1038/s41422-020-0287-8)
Supplement: Supplementary file 6 — Supplementary information, Figure S6 [file 41422_2020_287_MOESM6_ESM.pdf]

Supplementary information, Figure S6

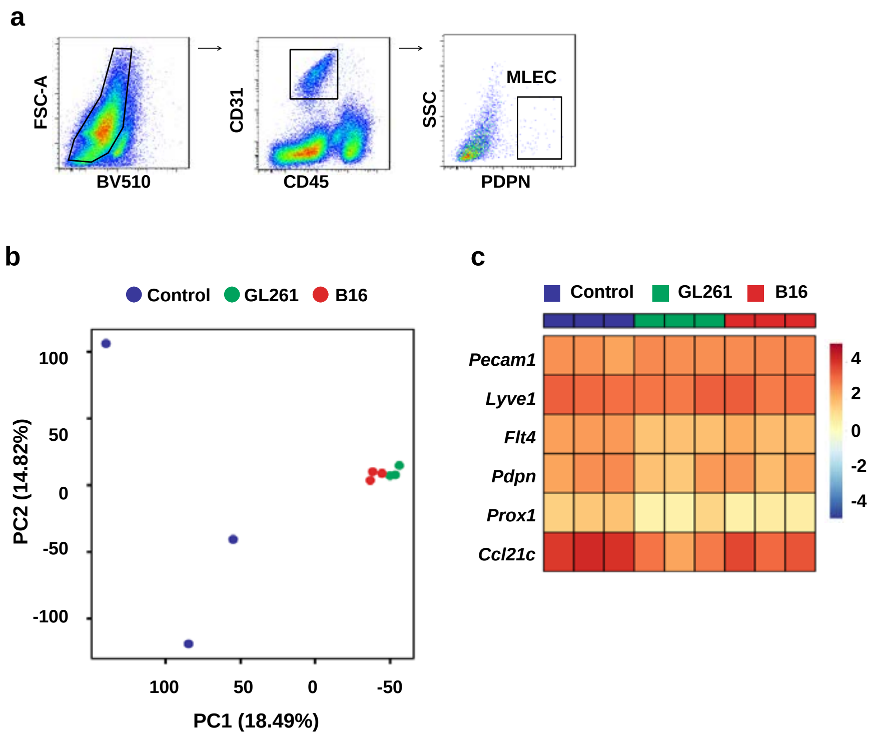

Fig. S6 No significant difference of marker gene expression in the MLECs isolated from the controls and tumor-bearing mice. a, Representative plots showing the gating strategy used to sort MLECs by FACS from control, GL261 tumor-bearing, and B16 tumor-bearing mice (n = 3). **b,** Principal component analysis of the transcriptome of control, GL261 tumor-associated, and B16 tumor-associated MLECs (n = 3, each pooled from 6 individual mice). **c,** Heatmap of LEC-related marker gene expression.
