# Supplementary material for: Meningeal lymphatic vessels regulate brain tumor drainage and immunity
Source: Cell Res. 2020 Feb 24;30(3):229–43. doi: 10.1038/s41422-020-0287-8 (PMC7054407; doi:10.1038/s41422-020-0287-8)
Supplement: Supplementary file 7 — Supplementary information, Figure S7 [file 41422_2020_287_MOESM7_ESM.pdf]

Supplementary information, Figure S7

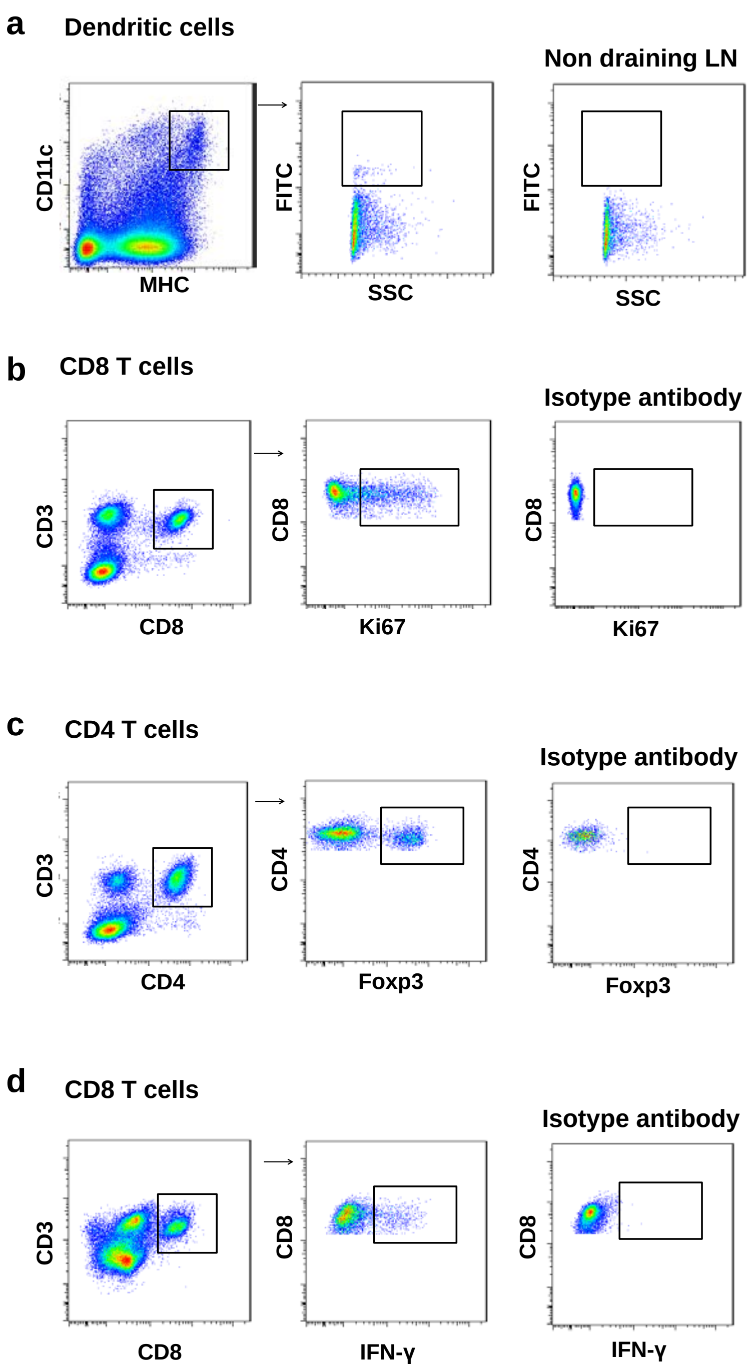

Fig. S7 Flow cytometry gating strategies of DCs and T cells. a-d, Representative contour plots for Bead$^+$ DCs (**a**), CD8$^+$Ki67$^+$ T cells (**b**), CD4$^+$Foxp3$^+$ T cells (**c**), and CD8$^+$IFN-γ$^+$ T cells (**d**). Cells were fixed using the Foxp3 kit and analyzed by flow cytometry for the lymphocyte markers, Ki67, IFN-γ. and Foxp3.
