# Supplementary material for: Meningeal lymphatic vessels regulate brain tumor drainage and immunity
Source: Cell Res. 2020 Feb 24;30(3):229–43. doi: 10.1038/s41422-020-0287-8 (PMC7054407; doi:10.1038/s41422-020-0287-8)
Supplement: Supplementary file 8 — Supplementary information, Figure S8 [file 41422_2020_287_MOESM8_ESM.pdf]

Supplementary information, Figure S8

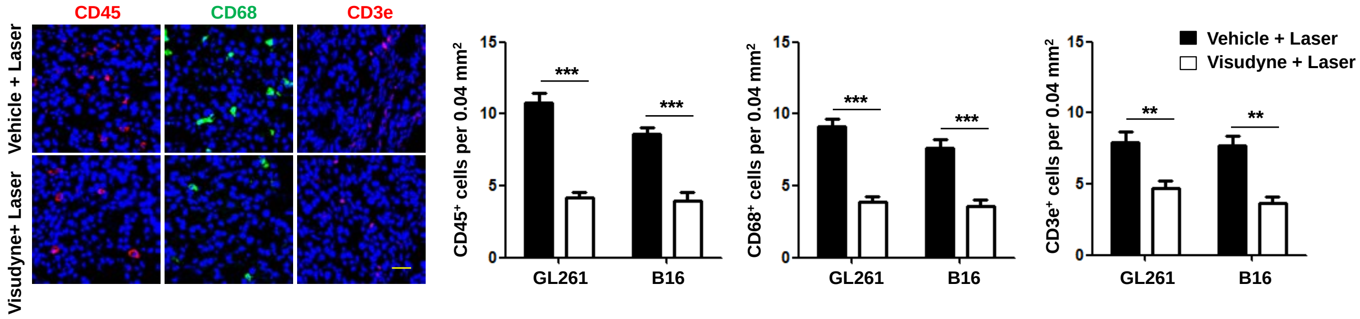

Fig. S8 Ablation of MLVs decreases tumor inflammatory infiltration. Representative sections of GL261 tumors showing DAPI, CD45, CD68, and CD3e staining from mice treated with Vehicle + Laser or Visudyne + Laser (left panel), and quantification of $CD45^+$, $CD68^+$, and $CD3e^+$ cells within GL261 and B16 tumors (right panels) (n = 10). Scale bar, 50 μm. Data are presented as the mean ± SEM. **P <0.01, ***P <0.001; two-way ANOVA. Data are from at least two independent experiments.
