# Supplementary material for: Meningeal lymphatic vessels regulate brain tumor drainage and immunity
Source: Cell Res. 2020 Feb 24;30(3):229–43. doi: 10.1038/s41422-020-0287-8 (PMC7054407; doi:10.1038/s41422-020-0287-8)
Supplement: Supplementary file 9 — Supplementary information, Figure S9 [file 41422_2020_287_MOESM9_ESM.pdf]

Supplementary information, Figure S9

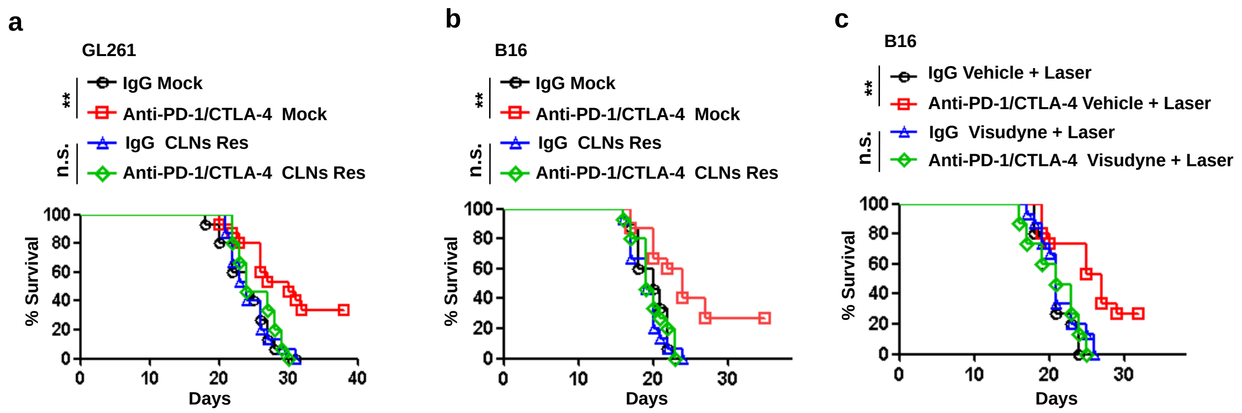

Fig. S9 CLN resection impairs the efficacy of striatally tumor immunotherapy. a, b, Survival of mice with striatally GL2161 tumors (**a**) and B16 tumors (**b**) following the administration of anti-PD-1/CTLA-4 or IgG controls (n = 15). Res, Resection (performed one day before tumor injection). **c,** Survival of MLV-defective or intact mice with striatal B16 tumors following the administration of anti-PD-1/CTLA-4 or IgG controls (n = 15). Data are presented as the mean ± SEM. **P <0.01, n.s. not significant; long-rank (Mantel-Cox) test (**a-c**). Data are from at least three (**a-c**) independent experiments.
