# Supplementary material for: Meningeal lymphatic vessels regulate brain tumor drainage and immunity
Source: Cell Res. 2020 Feb 24;30(3):229–43. doi: 10.1038/s41422-020-0287-8 (PMC7054407; doi:10.1038/s41422-020-0287-8)
Supplement: Supplementary file 10 — Supplementary information, Figure S10 [file 41422_2020_287_MOESM10_ESM.pdf]

Supplementary information, Figure S10

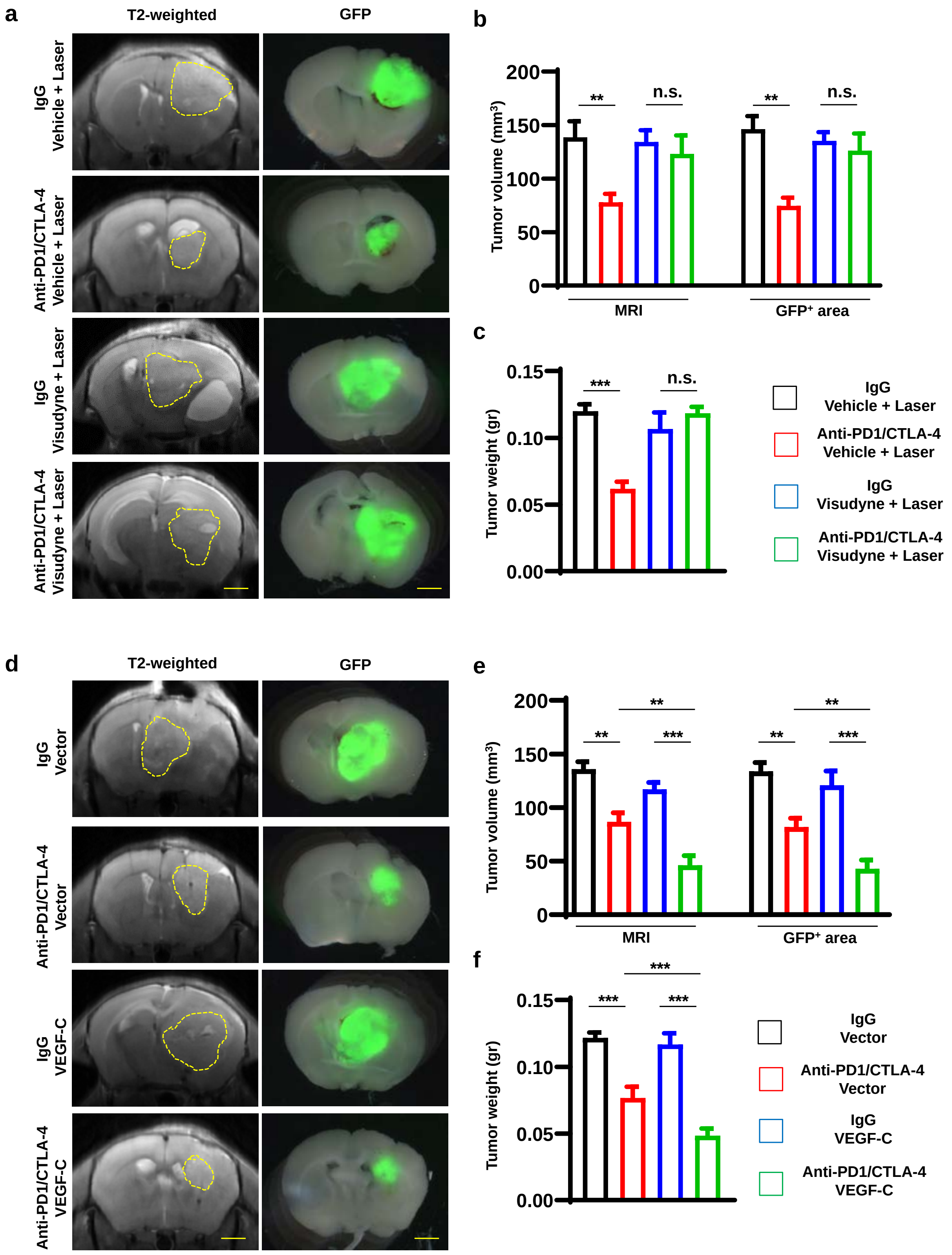

Fig. S10 Quantification of tumor volume and weight. **a**, Representative T2-weighted single brain slices (left panels) and GFP^+ areas (right panel) of tumor area in MLV-intact and MLV-defective mice bearing striatal GL261-GFP^+ tumors (*n* = 6). **b**, Quantification of tumor volume in **a**. **c**, Tumor weight in MLV-intact and MLV-defective groups (*n* = 6). **d**, Representative T2-weighted single brain slices (left panels) and GFP^+ areas (right panel) from mice with intracranial injection of GL261-GFP^+ cells overexpressing Vector or VEGF-C (*n* = 6). **e**, Quantification of tumor volume in **d**. **f**, Tumor weight in overexpressing Vector and VEGF-C groups (*n* = 6). Dashed lines indicate tumor margin. Scale bars, 3 mm. Data are presented as the mean ± SEM. $^{**}P<0.01$, $^{***}P<0.001$, n.s. not significant; two-way ANOVA. Data are from at least three (**a**, **d**) independent experiments.
