# Supplementary material for: Meningeal lymphatic vessels regulate brain tumor drainage and immunity
Source: Cell Res. 2020 Feb 24;30(3):229–43. doi: 10.1038/s41422-020-0287-8 (PMC7054407; doi:10.1038/s41422-020-0287-8)
Supplement: Supplementary file 11 — Supplementary information, Figure S11 [file 41422_2020_287_MOESM11_ESM.pdf]

Supplementary information, Figure S11

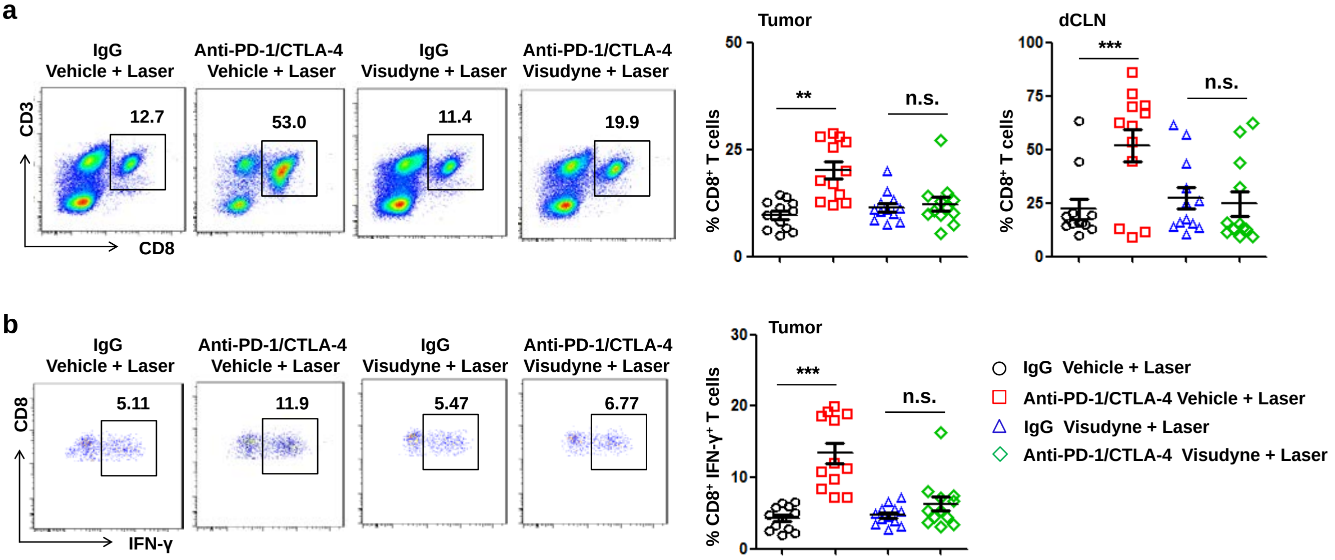

Fig. S11 Ablation of MLVs decreases the infiltration and IFN-γ production of $CD8^+$ T cells after anti-PD-1/CTLA-4 treatment. a, b, Representative examples (left) and quantification (right) of $CD8^+$ T cells (**a**) and $CD8^+IFN\text{-}\gamma^+$ T cells (**b**) as percentages of overall $CD45^+$ cells within dCLNs (*n* = 12 for each). Data are presented as the mean ± SEM. ***P* <0.01, ****P* <0.001, n.s. not significant; two-way ANOVA (**a**, **b**). Data are from at least three (**a**, **b**) independent experiments.
