# Supplementary material for: Meningeal lymphatic vessels regulate brain tumor drainage and immunity
Source: Cell Res. 2020 Feb 24;30(3):229–43. doi: 10.1038/s41422-020-0287-8 (PMC7054407; doi:10.1038/s41422-020-0287-8)
Supplement: Supplementary file 12 — Supplementary information, Figure S12 [file 41422_2020_287_MOESM12_ESM.pdf]

Supplementary information, Figure S12

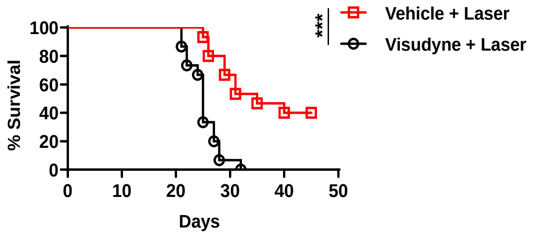

Fig. S12 Dorsal MLV ablation inhibites VEGF-C mediated enhancement of immunotheray. Survival of mice treated with Vehicle + Laser or Visudyne + Laser and striatally injected with GL261 VEGF-C overexpressing tumor following the administration of anti-PD-1/CTLA-4 antibodies (*n* = 15). ***P <0.001. Long-rank (Mantel-Cox) test.
